# Urine salts elucidate Early Neolithic animal management at Aşıklı Höyük, Turkey

**DOI:** 10.1126/sciadv.aaw0038

**Published:** 2019-04-17

**Authors:** J. T. Abell, J. Quade, G. Duru, S. M. Mentzer, M. C. Stiner, M. Uzdurum, M. Özbaşaran

**Affiliations:** 1 Lamont-Doherty Earth Observatory, Columbia University, New York, NY 10964, USA.; 2 Department of Earth and Environmental Sciences, Columbia University, New York, NY 10027, USA.; 3 Department of Geosciences, University of Arizona, Tucson, AZ 85721, USA.; 4 Department of Prehistory, Istanbul University, Istanbul 34134, Turkey.; 5 Mimar Sinan Fine Arts University, Istanbul 34134, Turkey.; 6 School of Anthropology, University of Arizona, Tucson, AZ 85721, USA.; 7 Institute for Archaeological Sciences, Eberhard Karls University of Tübingen, Tübingen 72070, Germany.; 8 Senckenberg Centre for Human Evolution and Paleoenvironment, Tübingen 72070, Germany.

## Abstract

The process of sheep and goat (caprine) domestication began by 9000 to 8000 BCE in Southwest Asia. The early Neolithic site at Aşıklı Höyük in central Turkey preserves early archaeological evidence of this transformation, such as culling by age and sex and use of enclosures inside the settlement. People’s strategies for managing caprines evolved at this site over a period of 1000 years, but changes in the scale of the practices are difficult to measure. Dung and midden layers at Aşıklı Höyük are highly enriched in soluble sodium, chlorine, nitrate, and nitrate-nitrogen isotope values, a pattern we attribute largely to urination by humans and animals onto the site. Here, we present an innovative mass balance approach to interpreting these unusual geochemical patterns that allows us to quantify the increase in caprine management over a ~1000-year period, an approach that should be applicable to other arid land tells.

## Teaser

Soluble salts in archaeological sediments reveal an increasing scale of caprine management at Aşıklı Höyük, Turkey.

## INTRODUCTION

**Fig. 1:**
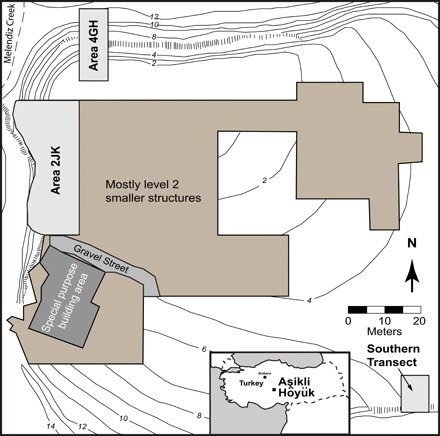
Map of Aşıklı Höyük excavation. Map showing excavation areas and sampling locations at Aşıklı Höyük. Three areas of sampling mentioned in the text (light gray areas) include area 4GH, area 2JK, or the west wall, and southern transect. Modified from Quade *et al*. (*8*).

The transition from hunting and gathering to farming and herding is thought to have occurred between 9000 and 6500 BCE in Southwest Asia, during the Pre-Pottery Neolithic. Human management of caprines (sheep and goats), along with pigs and eventually cattle, is one of the first manifestations of this socioeconomic change in Southwest Asia (*1*–*3*), alongside the domestication of cereals and pulse legumes. Aşıklı Höyük (*4*–*6*) in eastern Central Anatolia (Fig. 1, inset) preserves early evidence for human manipulation of sheep and goats by 8450 BCE (*7*, *8*) and a local evolution of these practices over the next 1000 years (ca. 8450 to 7450 BCE).

Aşıklı Höyük resides on a widened stretch of the Melendiz River, where rich soils suitable for plant cultivation had developed on Late Pleistocene marsh deposits (*9*). The settlement became permanent within just a few generations (*6*). A local process of wheat domestication ensued there (*10*), and the inhabitants experimented with propagating caprines from an early date (*7*). The first human occupations at Aşıklı Höyük (Level 5) were situated directly on the natural floodplain alluvium (*6*). By the latest period (Level 2), the tell covered an area of ~57,000 m^2^ and rose ~16 m above the floodplain of the Melendiz River. Erosion by the river meander and 30 years of archaeological excavation (*4*, *5*) have created exceptional vertical and lateral exposures of the tell’s deep archaeological layers and the underlying natural alluvium (Fig. 1). Levels 5 and 4 span ca. 10,400 to 10,000 calibrated years before the present (cal BP) (mid to late 9th millennium BCE), Level 3 spans 10,000 to 9700 cal BP (late 9th to early 8th millennium BCE), and Level 2 spans 9700 to 9300 cal BP (early to mid 8th millennium BCE) (*8*). From an originally broad and diverse diet in Level 5, peoples’ reliance on caprines and cultivated cereals and pulses increased gradually from Levels 4 to 2 (*7*, *10*, *11*).

The earliest occupation at Aşıklı Höyük in Level 5 may not have been fully sedentary, and the buildings and other structures were comparatively fragile. The residences were oval semisubterranean buildings constructed with wattle and daub and separated from one another by outdoor areas dotted with waste layers called middens, small enclosures, and work areas (*6*). Level 4 contains buildings of similar plan, but the walls were made of sun-dried mudbrick and are more massive. An architectural transition from oval semisubterranean buildings to aboveground quadrangular buildings occurs within Level 3, along with substantial growth of midden deposits and corralling areas. Level 2 shows marked consolidation and infilling of the architectural spaces on the mound, when animal enclosures seem to disappear from the mound top, but the overall dependence on caprine meat continued to increase. The ratio of indoor to outdoor space within the settlement was roughly equal in Levels 5 and 4 but shifted to greater indoor space by the time of Level 2, when more work was conducted indoors or on the high flat roofs of the residential buildings. Clustering of buildings also became prevalent during Level 2, with narrow alleyways separating dense architecture. Building clusters generated neighborhoods, and almost every neighborhood had its own midden area (*12*).

The bulk of the tell deposits consists of buildings and construction debris, predominantly mudbrick and mortar, and middens (*6*, *8*). The middens and small dumps contain varying mixes of sediment laced with animal bones, primary and charred plant matter, mudbricks and mortar, dung, wood ash, and a rich assortment of obsidian and other artifacts. Middens in Level 2 occur as (i) well-confined thick refuse concentrations, termed “communal middens,” where a wide range of activities such as butchering, animal corralling, and work also took place, and as (ii) diffuse dumping areas. Dump and midden deposits are least concentrated in Levels 4 and 5, where they are found mostly in the hollows of the abandoned semisubterranean buildings (*13*). The rate of refuse accumulation increased in the younger levels as the community grew in size and scale of mudbrick construction increased.

The site of Aşıklı Höyük provides a rare opportunity to examine the developmental subtleties of the first phases of a domestication process in a single location [(*14*) but see also (*7*, *8*, *15*, *16*)]. Human management of caprines is indicated by architectural remnants of wattle and daub corrals (*6*); micromorphological evidence of concentrated primary dung trampled in place by animals (*13*), often inside discernible enclosures; zooarchaeological evidence of selective culling of young male caprines before the age of 6 to 7 months (*7*); and phytolith, macrobotanical, and isotope evidence of foddering on site (*10*, *17*, *18*). The practice of keeping sheep and goats in captivity began on a very small scale in Level 5, and archaeological evidence indicates that it increased with time. The inhabitants’ economic dependence on caprines relative to other meat sources increased from about 26% of all animal remains in Level 5 to as high as 92% in the later phases of Level 2 (*7*, *16*). Limb joint pathologies suggest that animals were overly confined during the periods represented by Levels 5 to 3 and less so by the time of Level 2 (*17*). Elevated rates of joint pathology imply that the animals were penned much of the time. Outland pasturing did not begin until late in the sequence (Level 2) but largely from April to November, as the region experiences heavy snow in winter. Night penning was likely practiced in all periods on account of the prevalence of leopards, bears, and wolves in the area (*7*) and the near or total absence of guard dogs.

**Fig. 2:**
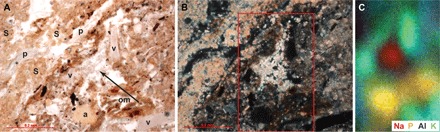
Secondary salts and mineral nodules in the micromorphology samples. (**A**) Photomicrograph of the contact between a layer of intact dung and the underlying sediment. The dung layer contains thin, undulating lenses of calcareous spherulites (S) interbedded with siliceous phytoliths (p). The sediment beneath contains fragments of volcanic glass (v), degraded organic material (om), and secondary nodules of apatite (a) in a matrix rich in wood ashes and clay minerals. A concentration of secondary mineral crystals—unidentified but likely soluble salts—formed within a void is indicated with an arrow. Plane-polarized light. (**B**) Same view as (A), cross-polarized light. Area scanned using micro–x-ray fluorescence indicated with the red box. (**C**) Elemental distribution map showing enrichment of sodium in the secondary mineral crystals, as well as phosphorus in the apatite and organic material, potassium in the volcanic glass, and aluminum in the clay-rich matrix. Note that although concentrations of suspected soluble salts (as well as other secondary minerals such as apatite and gypsum) have been observed in the micromorphology samples, further identification is limited because of highly variable crystal morphologies (*58*).

Zooarchaeological, paleobotanical, and architectural data from Aşıklı Höyük therefore show that human strategies for managing caprines underwent considerable evolution over a 1000-year period. However, changes in the scale of the practices have been very difficult to measure from these conventional archaeological data. In this study, we have developed a new and independent test for reconstructing the onset of and changes in the scale of stock-keeping with time based on the chemical composition of soluble salt in archaeological sediments, specifically urine-derived salts as proxies for the scope of metabolic activity on the mound. Micromorphological examination and Fourier transform infrared spectroscopy of middens and structural materials revealed the presence of numerous salts, including nitratine crystals (NaNO_3_), an unusual mineral that is typically found in extremely dry, saline environments (*19*) rich in Na^+^ and NO_3_^−^ (Fig. 2 and fig. S1). This mineralogical oddity first prompted us to explore the soluble salt (Na^+^, Cl^−^, NO_3_^−^, SO_4_^2−^, K^+^, Ca^2+^, and Mg^2+^) and nitrogen isotopic composition of the mound that might explain the presence of nitratine.

Here, we present a previously undescribed approach to interpreting the geochemical composition of an archaeological tell and its implications for early animal domestication. We measure the soluble salt composition of more than 100 samples as a function of material type, location in the mound, and age (table S1). For the purposes of this paper, we mainly focus on the patterns exhibited by [Na^+^], [Cl^−^], and [NO_3_^−^], which reach unusually high levels in the archaeological layers that are only partially explained by sources such as rainfall, wood ash, and natural sediment incorporated in building materials. In addition, we analyze nitrogen isotopes to identify the source of soluble nitrogen.

Our key finding is that urine—from ungulates and humans combined—provides the best explanation for the unusual mineralogy and salt composition of the tell deposits. We present a simple mass balance model that provides constraints on the scale of change in the number of caprines and humans that lived on the tell over ~1000 years of continuous occupation.

## RESULTS

### Soluble salt concentrations

We analyzed the soluble salts of 113 samples (see Methods) from three excavated areas of the tell: area 4GH or the “deep sounding” on the north face of the mound, area 2JK (or “west wall”) facing the Melendiz River, and lastly, the “southern transect” on the southeast side (Fig. 1). In general, [Na^+^], [Cl^−^], and [NO_3_^−^] (expressed as mean [] in moles × 1000 kg^−1^ ± 1σ SD) vary widely in sediments at Aşıklı Höyük, from very low levels in natural, nonarchaeological alluvium beneath the site to orders of magnitude increases (on average, with time) in the overlying archaeological strata. Of all the material types examined, general midden samples (*n* = 51) contain the highest [Na^+^] (4.44 ± 4.80), [Cl^−^] (6.86 ± 9.58), and [NO_3_^−^] (3.73 ± 8.32) (fig. S2 and table S2). Dung-rich and compacted dung midden samples (*n* = 9) also show elevated [Na^+^] (1.47 ± 2.08) and [Cl^−^] (1.61 ± 2.51) but much lower [NO_3_^−^] (0.105 ± 0.280) compared to average general midden. Samples from alleyways between buildings in Level 2 (*n* = 9) contain comparably high levels of all three ions. Construction debris composed of brick, plaster, and floor material samples (*n* = 9) contain soluble salt concentrations that range from ~2 to 50× less than general midden and dung-dominated samples (table S2). Last, samples from within and beneath hearths are chemically indistinguishable from dung-dominated midden. Midden samples, consisting of material from general midden, dung-dominated midden, and alleyways also exhibit notable spatial heterogeneity [1σ relative SD (RSD) of >200% of the mean in some cases] across horizontal units (Level 2 is used here as an example) in [Na^+^], [Cl^−^], and [NO_3_^−^] content (table S2 and fig. S3).

**Fig. 3:**
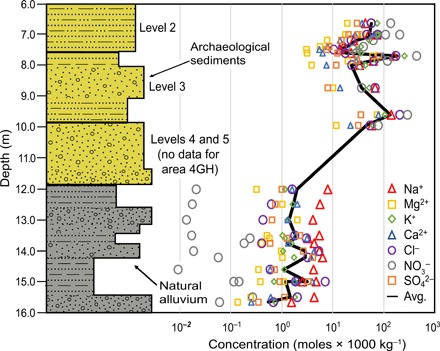
Stratigraphic profile and soluble salt concentrations from area 4GH (see Fig. 1 for location) at Aşıklı Höyük. (**Left**) Depth profile of the archaeological material and basal sediments, with specific major archaeological Levels 5 to 3 indicated. Alternating silt and sand represented by patterns on stratigraphic column, while grain size is shown through relative width of each section. (**Right**) Concentrations of seven soluble salts (see key) versus depth. The black curve denotes the average (Avg.) of all salts.

By contrast, the natural alluvium below level 5 in the west section (area 2JK, *n* = 8) and in area 4GH (*n* = 13) (Figs. 1 and 3) contains the lowest salt concentrations compared to all classes of archaeological material. [Na^+^] from subarchaeological alluvium in area 4GH (0.459 ± 0.167) and area 2JK (0.198 ± 0.125) is ~3 to 10× less than those found in hearths, dung-dominated layers, and construction debris and ~15 to 20× less than the alleyways and general midden samples. [Cl^−^] and [NO_3_^−^] are even lower compared to the archaeological material by a factor of ~2 to 60× and ~3 to 400×, respectively (table S2). Salt concentrations in the natural alluvium below the mound are also much more homogeneous than those in archaeological layers (1σ RSD of <150% of the mean) (table S2).

[Na^+^], [Cl^−^], and [NO_3_^−^] range widely, both vertically and laterally, within the archaeological levels, consistent with spatial segregation of activities in the settlement. For example, within Level 3, refuse [Na^+^], [Cl^−^], and [NO_3_^−^] vary vertically by a factor of ~5 among fine vertical layers in area 4GH (Fig. 3). Laterally, variability in [Na^+^], [Cl^−^], and [NO_3_^−^] within Level 2 can also differ by orders of magnitude, from generally much lower values in samples from area 2JK that are dominated by residential structures to higher levels in refuse-dominated locations in area 4GH and the southern transect (fig. S3). As an example, [NO_3_^−^] in area 4GH exhibits a mean of 9.77 ± 6.85, ~20 to 45× greater than those in area 2JK (0.522 ± 1.04) and the southern transect (0.204 ± 0.316).

**Fig. 4:**
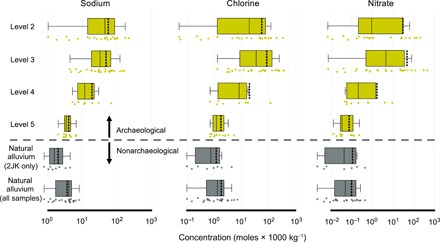
Comparison of soluble salt concentrations between archaeological and nonarchaeological sediments. (**A**) [Na^+^], (**B**) [Cl^−^], and (**C**) [NO_3_^−^] (in moles × 1000 kg^−1^) across major archaeological intervals (levels 5 to 2). Natural alluvium soluble salt concentrations from (i) all samples and (ii) samples directly beneath level 5 in area 2JK are provided only for comparison. Whisker lengths depict one-third of the interquartile range. The solid line within each box represents the median, while the dashed line shows the mean of the sample set. Data displayed here include general midden, dung-dominated midden, and alleyway samples combined.

At a coarser scale, averages of [Na^+^], [Cl^−^], and [NO_3_^−^] increase vertically through time from Levels 5 to 2 (Fig. 4). There is a 5 to 10× increase in [Na^+^], [Cl^−^], and [NO_3_^−^] from Levels 5 to 4 and an 10 to 1000× increase from Levels 5 to 3 (Fig. 4).

### Nitrogen isotopes

We isolated soluble NO_3_^−^ from 51 midden samples for δ^15^N analysis (table S1). Twenty-one midden samples and all but one basal natural alluvial sample [table S1, AHJQ-818-2 (IIIB)] lacked sufficient material after leaching (table S1) for isotope analysis. All midden samples returned δ^15^N_soluble_ values of [expressed as the population mean (μ), followed by the range] +13.2 per mil (‰), +5.8 to +17.7‰. Among these, samples of general midden (*n* = 41), dung-dominated midden (*n* = 3), and alleyways (*n* = 7) returned the highest δ^15^N_soluble_ (+13.2‰, +5.8 to +17.7‰; +14.2‰, +9.5 to +17.1‰; and +12.4‰, +9.1 to +17.2‰, respectively). Construction debris (*n* = 4) displayed a narrower and lower range of δ^15^N_soluble_ (+9.0, +7.0 to +11.2‰), as did hearths (*n* = 8; +8.2, +5.5 to +12.0‰). Nonarchaeological modern/Pleistocene floodplain sediments (*n* = 3) outside of the mound area, which were sampled to represent natural alluvium, yielded distinctly lower δ^15^N_soluble_ values (+5.4, +2.3 to +7.8‰).

## DISCUSSION

### Modeling the sources of soluble salts

#### Sources of soluble salts

The abundant soluble salts detected in nearly all the archaeological layers at Aşıklı Höyük most likely have multiple natural and anthropogenic sources, and our goal through construction of a simple mass balance model is to isolate the anthropogenic (human and livestock) component of salt that cannot be explained from other sources. The total concentration of a salt species *i* (*C*_Tot *i*_) (fig. S4A) in any given level at Aşıklı Höyük can be divided into those salts inherited from parent alluvial material (*C*_inherited *i*_) through incorporation into construction material, salts added anthropogenically (humans and captive animals) over the ~1000-year occupation of the mound (*C*_anthropogenic *i*_), and those added postdepositionally by rainfall (*C*_postdepositional *i*_) over a 10,000-year period, such that

$$C_{\text{Tot}\;i}=C_{\text{inherited}\;i}+C_{\text{anthropogenic}\;i}+C_{\text{postdepositional}\;i}$$ (1)

where *C* is the concentration of chemical species *i* expressed in moles per cubic meter in each component.

*C*_inherited *i*_ is composed of construction and building debris that is likely (at least initially) to be composed of the surrounding natural alluvium. The construction debris salt component (*C*_Tot cd *i*_) of the mound can be estimated on the basis of the chemical composition of the local, nonarchaeological alluvium. Excavations in 2015–2017 at Aşıklı Höyük penetrated ~1 to 2 m beneath the base of the tell over a broad area, exposing undisturbed floodplain sediments laid down before the first occupation of the site. These sediments are dominated by layered silt and mud with minor sand and diatomite. Our analysis reveals that the salt content of *C*_inherited *i*_ alluvial material is uniformly low, averaging ~11, 29, and 294× less in soluble Na^+^, Cl^−^, and NO_3_^−^, respectively, compared to archaeological refuse (all midden samples considered). The alluvium is thus distinctly low in its soluble salt composition (fig. S4B). This difference is readily visible in the large contrast between these elements across the archaeological/natural alluvial contact in the section from area 4GH (Fig. 3). In our calculations, we assume [Na^+^], [Cl^−^], and [NO_3_^−^] equal to the average (mean) values of natural alluvium for the construction debris (tables S2 to S5). These concentrations, with units of moles per kilogram, are converted to our workable concentrations (*C*_Tot cd *i*_) over a cubic meter by multiplying by the density of construction debris (table S6), determined in this study, and the fraction of midden composed of the weathered products of construction material (see the Supplementary Materials for explanation of adopted value). We do not use the concentration values of bricks and plaster measured for this study because many of these, especially in later levels, are created from recycled midden, which is likely the reason for their high soluble salt concentrations (fig. S2), and would thus not be indicative of inherited, local natural material.

*C*_anthropogenic *i*_ includes plant matter, bone, and wood ash. We can calculate the contribution of these components to *C*_anthropogenic *i*_ for a given chemical species *i* using their individual salt concentrations, their density, and their fractional contribution to total midden. The first two terms can be estimated from the literature (see the Supplementary Materials), and the third is determined in this study (see the Supplementary Materials). Wood ash, bone, and plants are major sources of SO_4_^2−^, PO_4_^3−^, Ca^2+^, Mg^2+^, K^+^, and CO_3_^2−^, but they contribute less to [Na^+^] (~63%), [Cl^−^] (<0.1%), and [NO_3_^−^] (~6.6%) inventories at the site (tables S3 to S5). Bone fragments were separated by dry sieving, and micrometer-scale decayed plant matter should contain little soluble salt. As such, these are not considered in the mass balance calculation. Wood ash (*C*_ash *i*_) is a large potential contributor of soluble salt, since it comprises ~25% of the tell refuse (see the Supplementary Materials). Combustion of wood creates an alkaline ash containing K^+^, lime, and other nutrients (*20*). Using an intermediate combustion temperature (*21*) of general wood ash (from wood and bark) that falls within the range of those determined from tree species near Aşıklı Höyük (*22*–*24*), the average content of wood ash–derived soluble salts can be calculated (tables S3 to S5) (*21*, *25*, *26*).

Postdepositional sources (*C*_postdepositional *i*_) of salts are delivered by rain and aerosols, which contain varying concentrations of solutes depending on closeness to the ocean and atmospheric circulation patterns (*27*, *28*). Industrial age increases in sulfur oxides and nitrogen oxides within atmospheric water should have little impact due to the short history of industrialization. To best represent the ~10,000 years of rain-borne solutes on the site, we used modern-day values from unpolluted locations in continental interiors globally for *C*_postdepositional *i*_ (also *C*_rain *i*_) (*28*). The total rain contribution to total soluble salt species (*C*_Tot rain *i*_) is calculated on the basis of the assumption of exponentially decreasing rainfall/salt deposition with soil depth, modeled after soil chloride data presented by Sandvig and Phillips (*29*). Additional variables are the time since occupation, the depth intervals of different archaeological levels, the annual precipitation rate, and an assumed runoff ratio.

Estimates for *C*_rain *i*_ were retrieved from the literature, as were the annual rainfall rate (*R*) of 0.4 m year^−1^ (tables S3 to S5). We selected a conservative rainfall runoff ratio (α) of 0.1, as this is the average for most sand/silt soil horizons on low slopes. We examine the treatment by the model of the runoff term and depth of penetration rainfall in more detail in the Supplementary Materials.

Up to this point, our modeling therefore assumes that *C*_inherited *i*_ = *C*_Tot cd *i*_, *C*_anthropogenic *i*_ = *C*_Tot ash *i*_ and *C*_postdepositional *i*_ = *C*_Tot rain *i*_. With estimates for all terms in our mass balance equation, we can calculate the remaining soluble salts within the mound not explained by our known sources (*C*_residual *i*_)

$$C_{\text{residual}\;i}=C_{\text{Tot}\;i}-(C_{\text{Tot cd}\;i}+C_{\text{Tot ash}\;i}+C_{\text{Tot rain}\;i})$$ (2)

This sums the contribution of all these depositional and known postdepositional components (*C*_Tot cd *i*_ + *C*_Tot ash *i*_ + *C*_Tot rain *i*_) and subtracts it from the total inventory of salts (*C*_Tot *i*_) (fig. S4A) to calculate that part of the salts unaccounted for (*C*_residual *i*_) (fig. S4C) by the depositional and postdepositional components of the tell considered so far. Using an average, we find that *C*_residual *i*_ is negligible for four of the soluble salts (SO_4_^2−^, Ca^2+^, K^+^, and Mg^2+^) because it can be reasonably explained by inputs from atmospheric and anthropogenic sources

$$C_{\text{Tot}({\text{SO}}_{4},\text{Ca},K,\text{Mg})}<\approx (C_{\text{Tot cd}({\text{SO}}_{4},\text{Ca},K,\text{Mg})}+C_{\text{Tot ash}({\text{SO}}_{4},\text{Ca},K,\text{Mg})}+C_{\text{Tot rain}({\text{SO}}_{4},\text{Ca},K,\text{Mg})})$$ (3)

In contrast, average soluble [NO_3_^−^], [Na^+^], and [Cl^−^] in archaeological layers are not nearly accounted for by atmospheric and anthropogenic sources

$$C_{\text{Tot}(\text{Na},\text{Cl},{\text{NO}}_{3})}\gg (C_{\text{Tot cd}(\text{Na},\text{Cl},{\text{NO}}_{3})}+C_{\text{Tot ash}(\text{Na},\text{Cl},{\text{NO}}_{3})}+C_{\text{Tot rain}(\text{Na},\text{Cl},{\text{NO}}_{3})})$$ (4)

We can express the proportions of *C*_residual *i*_ to *C*_Tot *i*_ fractionally as a percentage. Substituting $\left[C_{\text{Tot}({\text{Na},\text{Cl},\text{NO}}_{3})}\right]$ for species *i* in all depositional and known postdepositional terms in Eq. 2, we calculate that %_residual Na_ = ~25%, %_residual Cl_ = ~95%, and ${\%}_{\text{residual}\;{\text{NO}}_{3}}$ = ~88% (based on an average of general/dung-dominated midden and alleyway samples from all levels, which means specific levels may have higher or lower %_residual *i*_). The above calculations are for determining each component’s contribution to *C*_Tot *i*_ in moles per cubic meter. The sensitivities of model outcomes to uncertainties in the various components of the mass balance model are described in section S11.

#### The contribution from urine?

The proportions of Na^+^ (12%), NO_3_^−^ (27%), and Cl^−^ (61%) to total *C*_residual *i*_ deduced from our calculations bear a strong resemblance to the most abundant constituents, NO_3_^−^ (derived from total N), Na^+^, and Cl^−^, of human and caprine urine (fig. S4D and tables S3 to S5) (*30*, *31*), especially if our nitrate concentrations are corrected for ammonia volatilization. The elevated δ^15^N_soluble_ values (+13.2‰, +5.8 to +17.7‰) of the midden samples are unusual and provide critical supporting evidence for the urinary origins of N in tell refuse at Aşıklı Höyük. Nitrates and other salts are known to accumulate in arid land soils beneath the rooting depth of most plants, but δ^15^N values of these nitrates range from 0 to +10‰, too low to account for the higher values observed at Aşıklı Höyük (*32*, *33*). Moreover, salt concentrations at Aşıklı Höyük vary considerably on a fine scale of tens of centimeters from bed to bed, unlike the smoother decrease in salt concentrations with depth under natural arid land soils (*32*, *33*). High δ^15^N values can develop naturally in water-logged soils by denitrifying bacterial reduction of NO_3_^−^ (*34*, *35*), but there is no evidence of chemical reduction in the well-aerated and dry sediments composing the tell.

**Fig. 5:**
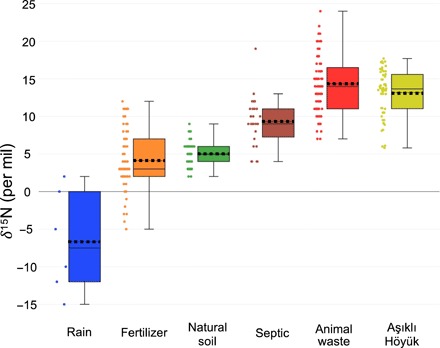
δ^15^N values (in per mil) of refuse samples from Aşıklı Höyük compared to a range of sources of nitrates. Whisker lengths denote one-third of the interquartile range. The solid line within each box represents the median, while the dashed line shows the mean of the sample set. Data not from Aşıklı Höyük are taken from (*39*–*43*).

High δ^15^N_soluble_ values of >10‰ are well documented from locations today where animal wastes and their breakdown products have been concentrated (*36*). For example, the highest biogenic δ^15^N values ever recorded (>+49‰) are found within penguin breeding areas in Antarctica (*37*); the elevated δ^15^N levels in the range of +7.4 to +13‰ are restricted to the soils and nearby ponds of the rookeries (*38*). Sediments underlying industrial animal feedlots provide a number of examples for the impact of animal waste on soil chemistry beneath the enclosures, well studied because of the contamination of local groundwater (*39*, *40*). There are strong similarities between high [NO_3_^−^] and δ^15^N_soluble_ values in Aşıklı Höyük’s refuse to that of reported data from five modern feedlots (*39*–*41*). Atmospheric rain and N_2_ fixation, fertilizer, natural soil, sewage, and animal waste additions to the feedlot system show δ^15^N ranges of −15 to +2‰, −5 to +12‰, +2 to +9‰, +5 to +19‰, and +7 to+ 26‰, respectively (Fig. 5) (*39*–*43*). Animal waste accounts for the highest δ^15^N values, an effect of the ammonia volatilization process discussed below. The range of δ^15^N_soluble_ values from Aşıklı Höyük falls closest to the mean of animal waste but also within the total range of human sewage (Fig. 5).

Soluble salt abundances under modern feedlots also resemble vertical patterns observed at Aşıklı Höyük. Fogg *et al*. (*40*) documented that over a depth of ~20 m, [Cl^−^] and [NO_3_^−^] under modern feedlots in California range between ~100 and ~1000 parts per million (ppm), markedly higher than only 1 to 50 ppm in natural soils outside feedlot areas. Similarly, [Cl^−^] and [NO_3_^−^] from general midden, dung-dominated midden, and alleyway samples at Aşıklı Höyük range from ~<1 to ~28,000 ppm (mean, ~1800 ppm) but only from ~<1 to ~250 ppm (mean, ~36 ppm) in natural alluvium beneath the site.

In summary, we conclude that in samples from general midden, dung-dominated midden, and alleyways, *C*_residual *i*_ ≈ *C*_urine *i.*_ based on the strong resemblance in salt geochemistry and δ^15^N_soluble_ values between strata underlying modern feedlots and that at Aşıklı Höyük.

Both human and sheep feces also contain all of the salts that have been discussed this far. However, dung is not considered in our mass balance calculation nor in our model used to estimate organism populations. One reason for this is that >99% of the total chlorine and >80% of the total sodium output for sheep are in urine (*44*). As such, it is unlikely that dung has contributed to more than ~20% of the total Cl^−^ and Na^+^. Nitrogen varies greatly in sheep dung and urine, and dung can contribute as much as ~50% of total nitrogen (*45*). However, the forms of nitrogen found in dung are less soluble than those of urine and may reduce the contribution of dung to the soluble NO_3_^−^ (*45*). This may explain why all salt concentrations in dung-dominated samples are lower than in general midden, especially if the dung in these layers is not in its original deposition location (fig. S2). Last, the highest [Na^+^], [Cl^−^], and [NO_3_^−^] are found in samples that do not have macroscale evidence of dung (general midden). For these reasons, we view dung as a minor contributor to our Na^+^, Cl^−^, and NO_3_^−^ totals and exclude dung in our calculations.

#### A closed system?

In the following section of this paper, our calculation of the number of organisms required to produce, through urination, *C*_residual Na_, *C*_residual Cl_, and $C_{\text{residual}\;{\text{NO}}_{3}}$ relies on the assumption that the mound geochemically acted as a closed system since deposition; i.e., it did not gain nor lose Na^+^, Cl^−^, and NO_3_^−^ postdepositionally. Our model calculations explicitly acknowledge that the mound at Aşıklı Höyük was not a perfectly closed system to infiltration by rainfall over the past 10,000 years. However, it should have been considerably less permeable than the great majority of open sites. Levels 2 and 3 capping the mound contain many plastered floors or other hardened occupational surfaces that would prevent or greatly reduce percolation of rainwater from above. Indirect evidence of limited percolation includes the very high quality of bone collagen preservation in Levels 2 and 3 used in paleo-DNA studies and ^14^C dating (*17*). The state of macroscopic organic preservation is even better in the lower parts of level 4 and in level 5 based on field observations.

Another potential closed system violation is loss of salts by leaching through the base of the tell. This possibility is contradicted by lower [Na^+^], [Cl^−^], and [NO_3_^−^], by approximately two orders of magnitude, (average, 111×) in the natural alluvium and abrupt increases in soluble salts above the contact between the alluvium and overlying archaeological layers. These patterns are consistent with modeling and observations from climatic settings in the southwestern United States similar to Aşıklı Höyük. Leaching depths of rainfall in these settings are confined largely to the upper ~2 to 3 m of soil profiles and fall off rapidly below 4 m (*29*). At Aşıklı Höyük, depths of 0 to 4 m fall almost entirely in Level 2, so we can expect little modification of primary anthropogenic geochemical patterns in Levels 3 to 5 and below by postdepositional rainfall and leaching. In addition, the values reported from other studies show elemental concentrations in both feedlot and natural areas (*40*) similar to those at Aşıklı Höyük. Ignoring potential closed system losses makes our estimates of the required urine fluxes conservative (i.e., minima). Physical erosion of the Aşıklı Höyük mound over the past ~10,000 years may seem like a violation of the closed system assumption, but erosion only introduces uncertainty in our estimates of total numbers of organisms, which relies on the assumption that the shape of the now partially eroded mound was originally circular.

Last, ammonia volatilization of nitrogenous compounds is probably a key process that would result in loss of total NO_3_^−^ and thus an underestimation of urination rates. Some N-rich compounds are lost after urination due to the process of ammonia volatilization:

1) (NH_2_)_2_CO + 2H_2_O → (NH_4_)_2_CO_3_

2) (NH_4_)_2_CO_3_ + 2H^+^ → 2NH_4_^+^ + CO_2_ + H_2_O

3) NH_4_^+^ + OH^−^ → NH_3_ + H_2_O

The higher proportion of nitrogen in liquid waste represents a combination of not only nitrate but also nitrite, urea, and uric acid concentrations. The amount lost depends on air temperature, percentage of soil moisture, soil porosity, plant uptake, relative humidity, and precipitation (*46*–*50*). Reynolds and Wolf (*46*) and Whitehead and Raistrick (*50*) both show an approximate loss of ~45% of urinary nitrogen in the form of ammonia under the climatic conditions of modern-day Turkey, which we adopt for our calculation of the corrected concentration of nitrate in urine ($C_{\text{urine}\;{\text{NO}}_{3}}$).

### Modeling of urination rates and Neolithic organisms

**Fig. 6:**
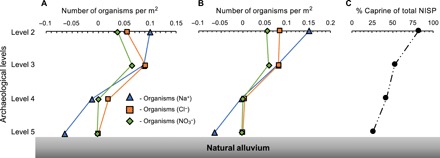
Model-predicted densities (organisms per square meter) of organisms required to produce urine-related [Na^+^], [Cl^−^], and [NO_3_^−^] found at Aşıklı Höyük, averaged across specific time intervals (levels 5 to 2). (**A**) Assuming a constant sedimentation rate over the entire period of occupation and (**B**) using variable sedimentation rates based on carbon-14–dated level boundaries (*8*). Densities of ≤0 indicate that conservative estimates of other inherited, anthropogenic, and postdepositional sources of soluble salts account for total [Na^+^], [Cl^−^], and [NO_3_^−^] observed in the refuse. (**C**) Relative abundance [percentage of the total number of identified skeletal specimens (NISP)] of caprines in the vertebrate faunal assemblages averaged by level [updated from (*7*) from Aşıklı Höyük].

We can now turn to estimating the density (*D*_org *i*_) of organisms per square meter for each archaeological Level (5 to 2) and the total number of organisms (*N*_org *i*_) for the entire tell, required to produce the calculated values of *C*_residual Na_, *C*_residual Cl_, and $C_{\text{residual}\;{\text{NO}}_{3}}$. Calculation of soluble [Na^+^], [Cl^−^], and [NO_3_^−^] produced by a single human and/or caprine assumes averages (*C*_urine *i*_) for these ions in urine of both groups taken from the literature [(*31*, *51*–*55*) and see tables S3 to S5 for full references], sedimentation rate, runoff fraction, urination rate, and fraction of time spent on the mound. We can then estimate the population density (*D*_org *i*_, in organisms per square meter) necessary to produce *C*_residual *i*_

$$D_{\text{org}\;i}=C_{\text{residual}\;i}/((C_{\text{urine}\;i}\times U_{R}\times (1-\alpha )\times F_{\text{om}})/\Gamma )$$ (5)

where α is the unitless runoff ratio, *U*_R_ is the urination rate (in liters per year), Γ is the sedimentation rate (in meters per year) (*8*), and *C*_urine *i*_ is the concentration of the salt species in the organism’s urine in moles per liter (tables S3 to S5). The sedimentation rate is determined in two different ways: (i) using a constant sedimentation rate throughout the entire mound and (ii) using dated level boundaries to define a sedimentation rate for each archaeological level [see tables S3 to S5 for values and (*8*) for dates and explanations]. Equation 5 yields estimates of increasing organism densities upward through the tell (Fig. 6, A and B, and tables S3 to S5), using both variable and constant sedimentation rates. Natural alluvium and Level 5 have comparably low-average [Na^+^], [Cl^−^], and [NO_3_^−^] (Fig. 4), suggesting that human/animal populations were initially very low, near background for preoccupation use of the area. *D*_org Na_ estimates are also near background for Level 4, but *D*_org Cl_ and $D_{\text{org}\;{\text{NO}}_{3}}$ increased to ~0.01 to 0.025 organisms per square meter in Level 4, and all three salt-derived organism densities jumped sharply to ~0.05 to 0.10 organisms per square meter in Level 3 (Fig. 6, A and B). Level 2 densities are comparable, slightly higher (in the case of *D*_org Na_) or slightly lower (in the case of *D*_org Cl_ and $D_{\text{org}\;{\text{NO}}_{3}}$) than Level 3. These estimates match closely with the changes in relative abundance of caprines in the vertebrate faunal assemblages averaged by level from Aşıklı Höyük (Fig. 6C) (*7*).

Last, we can use an average *C*_residual *i*_ (equal weighting of all four levels to avoid sampling biases) to calculate the average number of organisms that lived on the mound at any given moment over its ~1000-year duration by multiplying the produced average population density by the area (57,700 m^2^) of the tell

$$N_{\text{org}\;i}=D_{\text{org}\;i}\times A$$ (6)

Equation 6 yields an estimate of, on average, 1790 ± 510 (1σ SD) organisms that lived and urinated on the mound per day for the ~1000-year duration of the occupation based on all three soluble salts of interest.

Since animal and human urine are geochemically indistinguishable, this estimate cannot distinguish between animals and humans living on the site. However, it is likely that only humans and caprines were dominant contributors of urine to the mound deposits. Small rodents invaded the settlement in small numbers in all periods based on the presence of their skeletal remains (*7*) and coprolites (*10*). Postoccupation burrowing by other rodent species also occurred, mainly blind mole rats of the genus *Spalax*, but the low density of tunnels indicates that the number of resident animals would have been few. Dogs may have existed in this region and period, but traces of their presence are minimal or absent in the four levels.

## PERSPECTIVE AND CONCLUSIONS

An important but intractable question for archaeologists who study the forager-producer economic transition concerns the scale of human investment in animal management and the pace of its increases with time. This study uses urine salt inputs as a metabolic scale of the intensity of caprine management practices at Aşıklı Höyük by tracking, in relative terms, the growth of the community and its animals with each succeeding archaeological level.

Previous archaeological work at Aşıklı Höyük has shown that caprines were held captive and managed in small numbers inside the settlement from level 5 onward and that caprine management developed into a key part of the economy over the course of one millennium. What has been lacking, however, is reliable information about the scale of increase in biological (metabolic) activity on the mound, which we treat as a partial proxy of changing economic investments by the human inhabitants. Because some caprines were hunted rather than managed, especially in the earlier periods, independent evidence of the scope of captivity can be gleaned from the urine inputs from humans and livestock combined. At Aşıklı Höyük, the urine inputs greatly outstrip architectural evidence of human population density in each layer, as loosely indicated by the number of residential buildings (a topic of ongoing study) (*56*).

Five key outcomes of our study concern changes in human behavior as quantified by our new methodological approach. First, there are 5 to 10× increase in [Na^+^], [Cl^−^], and [NO_3_^−^] from levels 5 to 4 and 10 to 1000× increase from levels 5 to 3 at Aşıklı Höyük (Fig. 4). Second, urine inputs decline somewhat from levels 3 to 2, when higher architectural density and other data suggest that animal corrals were shifted to the mound periphery or areas of the mound that have yet to be excavated. Third, there is a marked spatial variation in urine inputs by humans and livestock in each layer, observations supported by micromorphological analyses of dung and midden (*57*). Middens and some alleyways must have been used as toilets by the humans. Animal urine accumulated not only wherever livestock were penned but also where humans used midden and dung as a binder in plasters and, probably more significantly, around fireplaces where humans recycled dung into fuel. The fourth outcome of the study is proof that simple techniques for determining abundances of major elements and δ^15^N values allow for the identification of urine as the dominant soluble salt contributor. The last outcome, also methodological, is that our approach can potentially be used to provide quantitative clues for animal management and/or human occupation in areas where there is a lack of other physical evidence (i.e., bones, dung layers, and major architecture). The analysis of urine salts as indications of metabolic activity in sites is only feasible; however, if chemical preservation in sediments is very good, such as in thickly stratified arid land tells with dense architectural features and in dry caves.

Returning to the larger questions posed by this research, the urine salt data demonstrate large increases in the scope and intensity of livestock keeping at Aşıklı Höyük over a span of 1000 years. The results contribute to evidence of a local (endemic) evolution of management practices. Aşıklı Höyük is located well outside (west of) the Fertile Crescent area, once believed to be the exclusive heartland of Neolithic emergence. Results such as ours demonstrate the existence of a much broader, diffuse network of societies involved in domestication processes and the evolution of Neolithic lifeways in Southwest Asia. The urine salt data put a scale to the evolutionary process at Aşıklı Höyük and thus represent a unique contribution in domestication research. Future studies involving estimates of human populations across archaeological levels will aid in distinguishing animal and human contributions to the soluble salt in archaeological deposits.

## METHODS

We analyzed 113 samples to develop a comprehensive spatial and temporal view of the soluble salt chemistry of the tell and areas surrounding Aşıklı Höyük. Fresh exposures on the north (area 4GH), west (area 2JK or “west section”), and south (southern transect) sides of the tell allowed us to sample laterally over tens of meters and vertically from the natural alluvium (24 samples) underlying the mound up through the major archaeological levels (89 samples; Figs. 1 and 3). Within the archaeological layers, general midden (*n* = 53), dung-dominated midden (*n* = 9), brick, mortar, and plaster (*n* = 7), hearths (*n* = 11), and alleyways (*n* = 9) were sampled (table S1).

All samples were analyzed for their major element composition and δ^15^N values in the soluble salt fraction. To isolate the soluble salts, 5 to 20 g of each sample was passed through a 5-mm sieve to remove coarse charcoal and plant matter, bone, obsidian flakes, hackberry endocarps, and gravel. Samples were then heated in 20–40 ml of distilled water (Milli-Q) at 70°C for 24 hours. The supernatant was decanted and set aside for soluble salt elemental analysis. A separate portion of the supernatant was evaporated in a drying oven for 48 hours. The evaporated salt residues were used for nitrogen isotope analysis, and the results are expressed in the familiar per mil notation as δ^15^N = [(*R*_sample_/*R*_air_) − 1] × 1000, where *R* = ^15^N/^14^N. Elemental analyses were conducted at the University of Arizona’s Laboratory for Emerging Contaminants housed in the Department of Soil, Water, and Environmental Science.

Anion concentrations were determined by ion chromatography on a Dionex ICS-1000 system with an anion exchange column (AG-22 + AS-22). Testing followed Method 4110 in Standard Methods for the Examination of Water and Wastewater, and the detection was completed using eluent conductivity and chemical suppression. Cations were analyzed using an Agilent 7700× ICP-MS (inductively coupled plasma mass spectrometry).

We calculated population means of [Na^+^], [Cl^−^], and [NO_3_^−^] in tell refuse to calculate population densities and numbers. Most of our sampling comes from middens, and the large sample size shows near log-normal to log-normal distributions (positive skew, due to the ~10^4^ range in values), where the mean and median are different by ~2 to 20×, depending on the ion. Since it is likely that the larger salt concentrations are indicative of organism urination and that the lowest values are likely midden samples devoid of urine from humans and caprines and/or areas not involved with the corralling of animals, means (averages) are the optimal values to use for estimating organism populations through our mass balance model, as means favor higher values in positively skewed populations. In addition, for salt concentrations in wood ash, rainfall, construction debris, and urine, we adopted average values from the literature and/or our study, so our usage of means for midden samples follows this procedure. As a result, we also reported means for all sample types in Results. An alternative is to use population medians, which would statistically better represent the positively skewed populations ([NO_3_^−^]). Use of medians generally yields ~2 to 4× lower paleo-organism estimates for Na^+^ and Cl^−^ and up to ~20× for NO_3_^−^ and shifts levels with currently (or close to) negative densities/populations farther negative. While this would not alter conclusions involving the relative changes in the scale of metabolic processes, it does introduce uncertainty in our absolute estimates. Tables S2 to S5 allow for the reader to better quantify changes in *C*_residual *i*_, *D*_org *i*_, or *N*_org *i*_ using medians instead of means. Future application of our model, using self-determined salt concentrations of urine, rainfall, wood ash, etc. at the site may be improved by implementing medians for all components, especially if the sampled populations of the other salt contributors and urine are nonnormal.

## Supplementary Material

http://advances.sciencemag.org/cgi/content/full/5/4/eaaw0038/DC1

Download PDF

Data file S1

## SUPPLEMENTARY MATERIALS

Supplementary material for this article is available at http://advances.sciencemag.org/cgi/content/full/5/4/eaaw0038/DC1 Section S1. Density and constituent fraction determination for midden and construction debris Section S2. Nitratine formation Section S3. Wood ash ion concentrations and density Section S4. Rainfall concentration and calculations Section S5. Runoff fraction for rain Section S6. Fraction of time spent on the site Section S7. Ion concentrations in human and caprine urine Section S8. Example calculation Section S9. Calculation of sedimentation rates Section S10. Heterogeneity of elemental concentrations across various samples Section S11. Sensitivity of the mass balance model Fig. S1. Infrared spectrum from a dung layer in midden (Level 3). Fig. S2. Comparison of salt concentrations in various archaeological and nonarchaeological materials. Fig. S3. Box and whisker plot of soluble salt concentrations (in moles × 1000 kg^−1^) across three sampling sections: area 4GH, area 2JK, and southern transect. Fig. S4. Four pie diagrams displaying soluble salt percentages. Table S1. Soluble salt chemistry and δ^15^N_soluble_ of archaeological and nonarchaeological layers at Aşıklı Höyük. Table S2. Statistical information of soluble salts based on material, spatial, and temporal setting. Table S3. Mass balance and organism estimation model of sodium at Aşıklı Höyük. Table S4. Mass balance and organism estimation model of chlorine at Aşıklı Höyük. Table S5. Mass balance and organism estimation model of nitrate at Aşıklı Höyük. Table S6. Density data from midden, construction material, and alluvium samples at Aşıklı Höyük. References (*59*–*81*)
