# Supplementary material for: Urine salts elucidate Early Neolithic animal management at Aşıklı Höyük, Turkey
Source: Sci Adv. 2019 Apr 17;5(4):eaaw0038. doi: 10.1126/sciadv.aaw0038 (PMC6469938; doi:10.1126/sciadv.aaw0038)
Supplement: http://advances.sciencemag.org/cgi/content/full/5/4/eaaw0038/DC1 [file supp_5_4_eaaw0038__index.html]

Science Advances | Science Advances

## Supplementary Materials

**The PDF file includes:**

- Section S1. Density and constituent fraction determination for midden and construction debris
- Section S2. Nitratine formation
- Section S3. Wood ash ion concentrations and density
- Section S4. Rainfall concentration and calculations
- Section S5. Runoff fraction for rain
- Section S6. Fraction of time spent on the site
- Section S7. Ion concentrations in human and caprine urine
- Section S8. Example calculation
- Section S9. Calculation of sedimentation rates
- Section S10. Heterogeneity of elemental concentrations across various samples
- Section S11. Sensitivity of the mass balance model
- Fig. S1. Infrared spectrum from a dung layer in midden (Level 3).
- Fig. S2. Comparison of salt concentrations in various archaeological and nonarchaeological materials.
- Fig. S3. Box and whisker plot of soluble salt concentrations (in moles × 1000 kg −1) across three sampling sections: area 4GH, area 2JK, and southern transect.
- Fig. S4. Four pie diagrams displaying soluble salt percentages.
- Table S1. Soluble salt chemistry and δ 15Nsoluble of archaeological and nonarchaeological layers at Aşıklı Höyük.
- Table S2. Statistical information of soluble salts based on material, spatial, and temporal setting.
- Table S3. Mass balance and organism estimation model of sodium at Aşıklı Höyük.
- Table S4. Mass balance and organism estimation model of chlorine at Aşıklı Höyük.
- Table S5. Mass balance and organism estimation model of nitrate at Aşıklı Höyük.
- Table S6. Density data from midden, construction material, and alluvium samples at Aşıklı Höyük.
- References (*59*–*81*)

Download PDF

**Other Supplementary Material for this manuscript includes the following:**

- Data file S1 (Excel format)

**Files in this Data Supplement:**

- Adobe PDF - aaw0038\_SM.pdf
