# Supplementary material for: Urine salts elucidate Early Neolithic animal management at Aşıklı Höyük, Turkey
Source: Sci Adv. 2019 Apr 17;5(4):eaaw0038. doi: 10.1126/sciadv.aaw0038 (PMC6469938; doi:10.1126/sciadv.aaw0038)
Supplement: Download PDF [file aaw0038_SM.pdf]

Science Advances
AAAS

advances.sciencemag.org/cgi/content/full/5/4/eaaw0038/DC1

Supplementary Materials for

Urine salts elucidate Early Neolithic animal management at Aşıklı Höyük, Turkey

J. T. Abell*, J. Quade, G. Duru, S. M. Mentzer, M. C. Stiner, M. Uzdurum, M. Özbaşaran

*Corresponding author. Email: jabell@ldeo.columbia.edu

Published 17 April 2019, *Sci. Adv.* **5**, eaaw0038 (2019)
DOI: 10.1126/sciadv.aaw0038

The PDF file includes:

Section S1. Density and constituent fraction determination for midden and construction debris
Section S2. Nitratine formation
Section S3. Wood ash ion concentrations and density
Section S4. Rainfall concentration and calculations
Section S5. Runoff fraction for rain
Section S6. Fraction of time spent on the site
Section S7. Ion concentrations in human and caprine urine
Section S8. Example calculation
Section S9. Calculation of sedimentation rates
Section S10. Heterogeneity of elemental concentrations across various samples
Section S11. Sensitivity of the mass balance model
Fig. S1. Infrared spectrum from a dung layer in a midden (Level 3).
Fig. S2. Comparison of salt concentrations in various archaeological and nonarchaeological materials.
Fig. S3. Box and whisker plot of soluble salt concentrations (in moles × 1000 kg^{-1}) across three sampling sections: area 4GH, area 2JK, and southern transect.
Fig. S4. Four pie diagrams displaying soluble salt percentages.
Table S1. Soluble salt chemistry and $\delta^{15}N_{soluble}$ of archaeological and nonarchaeological layers at Aşıklı Höyük.
Table S2. Statistical information of soluble salts based on material, spatial, and temporal setting.
Table S3. Mass balance and organism estimation model of sodium at Aşıklı Höyük.
Table S4. Mass balance and organism estimation model of chlorine at Aşıklı Höyük.
Table S5. Mass balance and organism estimation model of nitrate at Aşıklı Höyük.
Table S6. Density data from midden, construction material, and alluvium samples at Aşıklı Höyük.
References (*59–81*)

Other Supplementary Material for this manuscript includes the following:

(available at advances.sciencemag.org/cgi/content/full/5/4/eaaw0038/DC1)

Data file S1 (Excel Format)

Supplementary Methods and Text

Section S1. Density and constituent fraction determination for midden and construction debris

We measured the density of 20 samples from midden, alleyways, construction debris, natural alluvium (bulk soil), and a hearth sample (table S6). The unweighted mean of 15 midden/alleyway sample densities was ~1.4 g cm^{-3}, which is the value applied to all midden samples in our model. Construction debris density was also ~1.4 g cm^{-3}, which was determined from the unweighted average of two plaster samples and two bulk soil samples.

F_i, the fraction of total midden that is comprised of a specific component, was determined for both wood ash (F_{ash}) and construction debris (F_{cd}) in this study. Wood ash fraction was calculated based the percent carbonate ($\%_{Carb\ i}$) of the mound, with the assumption that all carbonate is originates from wood ash and that the ash-derived carbonate predominantly comes from midden and construction debris, such that

$$\%_{Carb\ mound} = ((\%_{Carb\ midden} * F_{mound\ midden}) + (\%_{Carb\ cd} * F_{mound\ cd}))$$

$$\%_{Carb\ mound} = ((17.5\% * 0.7) + (9.5\% * 0.3))$$

$$\%_{Carb\ mound} = \sim 0.15 \text{ or } 15\%$$

As such, the average carbonate percentage at Aşıklı Höyük is ~15%. However, wood ash is only ~55-65% carbonate (*21, 25*), so to obtain the total fraction of wood ash (F_{ash})

$$F_{ash} = \%_{Carb\ mound} / 0.6$$

$$F_{ash} = 15\% / 0.6$$

F_{ash} = ~0.25 or 25%

Assuming that ~4% of the midden is made up of bone, obsidian, and other larger constituents, then the remaining ~71% is construction debris (F_{cd}). We arrive at estimates of F_{ash} = 0.25, and F_{cd} = 0.71.

Section S2. Nitratine formation

[Na^+] and [NO_3^-] in the range of reported $C_{residual\ Na}$ and $C_{residual\ NO3}$ fall within the range reported from surface soils in the hyperarid regions of the world such as the Atacama (1.2 to 66 g/kg) and Death Valley, CA (1.8 to 28 g/kg) (*33*), where nitratine forms naturally (*19*). Nitratine requires relative humidity <~75.4% at 20°C and ~77.5% at 10°C to precipitate at the surface or near-surface (*59*), and as a salt, requires a very dry climate to be re-dissolved and leached. The site of Aşıklı Höyük is located in a drier region of Turkey and maintains an average relative humidity that ranges from ~49% to 62% (corresponding to average temperature maximums and minimums of 5.5°C to 18.4°C, respectively), dry enough to permit nitratine formation (*60*). Additionally, although Turkey is wetter than the Atacama and Mojave Deserts (200-500 mm/yr vs 1-60 mm/yr, respectively), the deeper vadose zone has probably remained closed to leaching during the Holocene (*60*-*63*). Paleoclimate records of hydrology and temperature do not show a large difference in southeastern Europe climate during the early Holocene, so near-modern climatic values can be safely assumed (*64*-*66*). In short, a large post-depositional flux of Na^+ and NO_3^- is necessary to form nitratine in the tell, combined with no salt losses through the base of the site because of its thickness and relatively dry Holocene climate.

Section S3. Wood ash ion concentrations and density

All elemental concentrations for wood ash are derived from (*21*) except for chlorine, which was not provided. A summary of the data collected from (*21*) and comparison to another study that focused on wood ash compositions can be found in (*25*). (*26, 67*) compiled some of the elemental concentrations relevant to our study for a) various tree species, and b) type of material burned, confirming that our selected values from (*21*) fell within error of the average of this compilation (*26, 67*). [Na^+] has been shown to vary between species and between components of the original live wood material (inner wood, bark, pulp, foliage, roots, etc.) that is burned (*26, 67*), but for species outside of poplar, tends to be quite low (*67*), much lower than our adopted value of $C_{ash\ Na}$. We use values from (*21*) of 3.4 g kg^{-1} for [Na^+] and 0.6 g kg^{-1} for [NO_3^-] because of their analysis of wood *and* bark ash. Chlorine, which was not measured in any of the cited studies, is a very minor constituent in wood and wood ash (*68*). Here we applied a value of 0.001 g kg^{-1} for [Cl^-], although this is likely an overestimate. Density of wood ash (ρ_{ash}) in our model is set to 0.760 g cm^{-3} (*69*).

Section S4. Rainfall concentration and calculations

[Na^+] in rainfall is derived from the literature based on a data from global inland continental sites that should not have been subjected to significant pollution, which can greatly affect [NO_3^-] and [Cl^-] (*28*). The total contribution of Na^+, NO_3^- and Cl^- from rainfall since initial occupation is calculated by multiplying the concentration of various salts in rain ($C_{rain\ i}$) by the yearly rainfall average (R, $m\ yr^{-1}$), the time since initial occupation (t, 10,000 years), and by the runoff ratio (α) (see following section for derivation) subtracted from 1. This value is the total flux of salt

species i to the 14 m of archaeological material over 10,000 years ($\Phi_{rain\ final\ i}$). This assumes all the Na^+, NO_3^-, and Cl^- accumulates uniformly over the 14 m of the tell profile.

A more realistic approach to assume exponentially decreasing atmospheric salt accumulation with soil depth from peak values at ~1.5-2.5m below the surface (*29*). Using modeled profiles of [Cl^-], which show a ~1/20 ratio of maximum [Cl^-] to those found at ~4 m depth, we develop an exponentially decreasing salt accumulation profile for Aşıklı (*29*). For our purposes, the peak in [Cl^-] found ~1.5-2.5m below the surface in the model derived from the literature is considered the top of Level 2 (this is unlikely as many of our samples from Level 2 were taken at least 3-4 m below the surface, but here we provide conservative estimates). Our equation for the flux of rainfall solutes per m^2 over the thickness of the mound for 10,000 years then becomes

$$\Phi_{rain\ final\ i} = C_{rain\ \ i} * R * (1-\alpha) * t = \int_0^{14} \Phi_{total\ rain\ i}(z)\, dz = (((\Phi_{rain\ o\ i}) / -k) * e^{-k*14})) - ((\Phi_{rain\ o\ i}) / -k) * e^{-k*0})))$$

Our initial known variables for this integration are the total integrated flux of salts within the 14 meters of tell accumulation over 10,000 years ($\Phi_{rain\ final\ i}$) and the depth range of 0-14 m. The integral of the exponential equation from 0-14 m is equal to the total flux of salts ($\Phi_{rain\ final\ i}$). We can then solve this equation for the surface flux ($\Phi_{rain\ \ o\ i}$) iteratively by changing k values so that the ratio of $\Phi_{total\ rain\ i}$(4m) to $\Phi_{rain\ i\ \ o}$ is ~1/20 (based on *29*). Our k value is determined to be -0.75 for all three soluble salts. The associated $\Phi_{rain\ i\ \ o}$ can be found in supplementary tables S3-S5.

With all variables of our initial equation known, we can integrate $\Phi_{total\ rain\ i}(z)$ over our depth ranges of interest ($\Phi_{rain\ level\ i}$)

$$\int_{z_1}^{z_2} \Phi_{rain\ level\ i}\ (z)\ dz\ = (((\Phi_{rain\ o\ i} / -k) * e^{-k*hB}) - ((\Phi_{rain\ o\ i} / -k) * e^{-k*hT}))$$

where h_B and h_T are the height of the top and bottom of each level. In our case, we assign 5m, 4m, 3m, and 2m as the thicknesses of Levels 2, 3, 4, and 5, respectively.

Finally, we divide this flux of salts at each level over 10,000 years by the thickness of the level to obtain the concentration of salts on a cubic meter basis ($C_{Tot\ rain\ i}$)

$$C_{Tot\ rain\ i} = \int_{z_1}^{z_2} \Phi_{rain\ level\ i}\ (z)\ dz\ /\ (h_B - h_T)$$

We conclude that 95% of the salts from rainfall over 10,000 years accumulated in Level 2.

Section S5. Runoff fraction for rain

Runoff is the fraction that runs off rather than infiltrates the soil after a rainfall event, and is defined as the runoff coefficient, α. α depends on a range of factors but generally increases as rainfall increases (*70*), because frequently wetted soils reject infiltration. Other variables effecting α include soil texture, vegetation cover, surface roughness, temperature, and slope. Aşıklı is partially eroded today by lateral erosion by Melendiz Creek. Through most of the last 10,000 years, extant remains suggest that the original site was a roughly conical shape, 14 m thick and nearly flat at the top, with smooth flanks sloping 10-20° in all directions. The surface of the site is underlain by a shallow silty soil, and is moderately vegetated with grasses and low-lying shrubs and herbs.

Settings in similar Mediterranean climate settings (MAP~0.3-0.7 m/yr, winter rainfall, silty soils, grass-dominated) in Spain (*71*), California (*72*), and Israel (Tel Melha watershed, data from Israel Meteorological Service (Beit Qama gauging station) and Israel Hydrological Service) experience ~10±5% runoff of their mean annual rainfall, or α ~0.1. This is the figure we adopt for our model calculations, but it is certainly conservative (minimizes our paleo-population estimates) given three factors at Aşıklı: the high slopes, patchy, degraded vegetation, and extensive plastered floors of Level 2 capping the site, all of which would tend to decrease infiltration and increase run off from the site (*73*).

Section S6. Fraction of time spent on the site

Our modeled value of urine salts delivered to the tell are also dependent upon the fraction of a 24-hour period that the organisms spend on the mound (F_{om}), as this is when they were likely to urinate within the confines of our sampling. We have already provided evidence for the confinement of caprines on-site for most of the time during Levels 4 and 5, and for increased daytime pasturing in the more recent periods of occupation (*17*).

Humans also contributed urine to the deposits, and they could have ranged more freely than livestock both on and off the mound. Much of the space within the village was devoted to domestic activities of all sorts. Ethnographic studies have shown that the domestic environment and debris accumulation therein are dominated women, children and the elderly (e.g., *74*). The activities of adult men and adolescent boys generally are more wide ranging in the surrounding landscape. Women's work would include garden tending and travelling to collect wild plant foods, firewood and raw materials, and general heavy lifting. In fact, skeletal remains of many of the Aşıklı women showed signs of traumatic arthritis in the neck and back vertebrae, suggesting

that they were carrying heavy loads (*75*). All of these pieces of evidence point to potential off-site work. On the other hand, many of the time-intensive tasks that women must have performed daily at Aşıklı Höyük included childcare, weaving plant fibers into mats and containers, preparing and grinding grain, cooking, processing animal carcasses, and cleaning and tanning hides, which are on-site activities. It is likely that shifts in the on-site-to-off-site time spent on the tell for men and women also occurred as time progressed.

As a conservative estimate for our model, we assume that during most daylight hours (when the majority of daily urination occurs), caprines and humans were offsite (a Level 2-centric perspective), grazing and working, respectively, while at night both remained onsite. Thus, the fraction of nighttime hours to the total 24-hour period of a day would be when the accumulation of salts occurs. Based on latitude, Turkey receives an annually averaged value of ~12 hours a day. This means that F_{om} is equal to 0.5, the value we tentatively assume in our model.

Section S7. Ion concentrations in human and caprine urine

Tables S3-S5 list the averages for [Na^+], [Cl^-], and [NO_3^-] of humans and caprines to estimate population densities and overall populations of organisms at Aşıklı. Here we describe how these concentrations were obtained from the literature.

A) Human Urine Concentrations

Nitrogen concentrations [N] for human urine were determined by taking the unweighted average of values listed from (*51, 76-78*). These were chosen because these studies sampled urine directly from the source (humans), before decomposition of major constituents such as urea occur, which results in the loss of nitrogen (*30, 52*). We use the value for total [N] corrected for

nitrogen loss, which assumes that ~55% of the nitrogen is converted to nitrate, while the remainder is lost during ammonia volatilization (*47, 51*). [Cl^-], [Na^+], [SO_4^{2+}], [K^+], [Ca^{2+}], and [Mg^{2+}] are taken from (*30*), which sampled urine from separated sewage from two cities in the Netherlands. Unlike NO_3^-, these ions should not be lost to volatilization with time.

B) Caprine Urine Concentrations

Estimates of [Na^+], [N], [SO_4^{2+}], [K^+], [Ca^{2+}], and [Mg^{2+}] for caprine urine are derived from (*31*), which collected fresh urine from four Scottish ewes. As there is little data available on [Cl^-] in sheep or goat urine, we use estimates from (*45*) to derive an approximate [Cl^-]. We calculate total cations (based on K^+ making up ~65% of the total, *45*), and multiply this number by 0.35, as it is assumed that Cl^- is responsible for ~35% of the anion total (*45*).

C) Conversion to Yearly Flux of Salt Ions

It has been shown in many previous studies that there is large variability in both caprine and human urine ion concentrations (*31*, *77*, *79*). However, it is likely the actual ionic flux per day of material is similar between organisms across the globe in order to maintain salt balances within the body. This has been shown for sheep and humans (see work referenced in *45, 52*). This implies variability in the urination amount per urination event of organisms per day to compensate. With this in mind, we must use an applicable daily urination rate for our human and caprine ion concentrations. On average, humans produce 1-1.5 liters of urine a day (*77, 80*). To this end, we assume a 1.25 L day^{-1} urination rate for humans. For caprines, we use estimates of urination events per day (17.5 event day^{-1}) and total liters per event (1.0 L $event^{-1}$) of sheep from a study (*81*) in Great Britain, close to the work of (*31*), to develop a value for our daily urination

rate (U_R). (*31*) did not provide values for the daily urination rates of the ewes in their study, which is the reason behind using values from (*81*).

Section S8. Example calculation

For transparency and ease-of-use, we present a step-by-step example of the application of our mass balance model, in this case showing how the organismal density and total number of organisms present at Aşıklı during Level 2 was calculated using our model based on the [NO_3^-] in Level 2 midden samples.

A) Conversion from laboratory output (ug/L) to mol/kg

Initially, data received from the laboratory is reported in ug/L ($C_{init\ NO3}$). This is then converted to the total mass of nitrate (m_{NO3}) in the water sample by multiplying the ug/L by the total amount of water used for the extraction (V_{water})

1) $m_{NO3} = C_{init\ NO3} * V_{water}$

This resulting mass of NO_3^- with units of ug is then divided by the mass of the total sample leached (m_{sample}, kg), and converted to the final workable [NO_3^-] (C_{NO3}) with units of mol kg^{-1}, by dividing by $1x10^6$ and by the molar mass of nitrate (M_{NO3}, 35.45 g mol^{-1}) for all general midden, dung-dominated midden, and alleyway samples in Level 2 to produce a final average

2) $C_{NO3} = (m_{NO3} / m_{sample}) / (1x10^6 * M_{NO3})$

B) Total [NO_3^-] in mol m^{-3}

We begin by taking the raw unweighted average molar [NO_3^-] (C_{NO3}, mol kg^{-1}) of 22 midden samples, which includes general midden, dung-dominated midden, and alleyways, from all of Level 2, that is: C_{NO3} = 3.09E-02 mol kg^{-1} (table S2). From this we can calculate the molar [NO_3^-] of total NO_3^- per cubic meter ($C_{Tot\ NO3}$) in the tell refuse by multiplying the average [NO_3^-] in the samples (C_{NO3}) by the average density of midden (ρ_{refuse} = 1.4 g cm^{-3} = 1400 kg m^{-3})

3) $C_{Tot\ NO3} = C_{NO3} * \rho_{refuse}$

A) $C_{Tot\ NO3}$ = 3.09E-02 mol kg^{-1} * 1400 kg m^{-3}

$C_{Tot\ NO3}$ = 43.22 mol m^{-3}

C) Accounting for Various Sources of NO_3^-

Total [NO_3^-] ($C_{Tot\ NO3}$) in Level 2 calculated above likely has both natural and human-related sources:

Wood ash. We calculate the wood ash NO_3^- contribution ($C_{Tot\ ash\ NO3}$, mol m^{-3}) in a cubic meter of refuse by assuming the average [NO_3^-] derived from literature in wood ash ($C_{ash\ NO3}$) and multiplying by the average density of the wood ash (ρ_{ash}) and a conservative estimate of total refuse volume composed of ash (F_{ash} = 0.25)

4) $C_{Tot\ ash\ NO3} = C_{ash\ NO3} * \rho_{ash} * F_{ash}$

B) $C_{Tot\ ash\ NO3}$ = 1.00E-02 mol kg^{-1} * 760 kg m^{-3} * 0.25

$C_{Tot\ ash\ NO3}$ = 1.84 mol m^{-3}

Inherited NO_3^- in parent material. Inherited soluble NO_3^- includes the incorporation of natural sediments beneath and around the archaeological site, mostly fluvial sands and silts, into building materials, such as brick and mortar. To account for the associated NO_3^- contribution ($C_{Tot\ cd\ NO3}$) from construction debris to the total ($C_{Tot\ NO3}$), we calculate the [NO_3^-] in mol m^{-3} by multiplying the average [NO_3^-] in the building materials ($C_{cd\ NO3}$), which is based on an unweighted average of the 21 natural alluvium samples taken beneath the mound (table S2), by the average density (ρ_{cd} = 1400 kg m^{-3}) and by the estimated fraction of building material (F_{cd} = 0.71) in refuse

5) $C_{Tot\ cd\ NO3} = C_{cd\ NO3} * \rho_{cd} * F_{cd}$

C) $C_{Tot\ cd\ NO3}$ = 9.53E-05 mol kg^{-1} * 1400 kg m^{-3} * 0.71

$C_{Tot\ cb\ NO3}$ = 0.090 mol m^{-3}

Precipitation. A final source of all salts including NO_3^- is that dissolved precipitation (rain and snow) that fell and infiltrated the site during the ~10,000 years since initial occupation. To calculate the contribution from precipitation ($C_{Tot\ rain\ NO3}$), we multiply [NO_3^-] in rain ($C_{rain\ NO3}$) for an average of unpolluted areas in the inner regions of continents (8.06E-06 mol L^{-1}, *27*) by the mean annual rainfall received per year (R, 0.4 m yr^{-1} = 400 L m^{-2} yr^{-1}, *60*). This is then multiplied by a runoff ratio (α) subtracted from 1, which has been assigned a value of 0.1 as a conservative estimate for this region and by the time since initial occupation (t, 10,000 years). This yields a precipitation-derived NO_3^- flux per square meter over a ten thousand-year period ($\Phi_{rain\ final\ NO3}$). We use this flux to derive the k and $C_{rain\ o\ NO3}$ values for our exponential function

(see tables S3-S5 and supplement section 4). To derive the final molar [NO_3^-] per cubic meter from rainfall in Level 2, we divide our integrated flux by the thickness of Level 2 ($h_B - h_T$)

6)

$$\Phi_{rain\ final\ NO3} = C_{rain\ NO3} * R * (1-\alpha) * t = \int_0^{14} \Phi_{total\ rain\ NO3}\ (z)\ dz = (((\Phi_{rain\ o\ NO3}) / -k)*e^{-k*14}) - ((\Phi_{rain\ o\ NO3}) / -k)$$

(solve for k and $\Phi_{rain\ o\ NO3}$ iteratively, which are -0.75 m^{-1} and 21.77 mol m^{-2}, respectively)

7) $\int_0^{5} \Phi_{total\ rain\ NO3}\ (z)\ dz = (((\Phi_{rain\ o\ NO3} / -k) * e^{-k*hB}) - ((\Phi_{rain\ o\ NO3} / -k) * e^{-k*hT}))$

$\int_0^{5} \Phi_{total\ rain\ NO3}\ (z)\ dz$ = 28.35 mol m^{-2} (see tables S3-S5)

8) $C_{Tot\ rain\ NO3} = \int_0^{5} \Phi_{total\ rain\ NO3}\ (z)\ dz / (h_B - h_T)$

D) $C_{Tot\ rain\ NO3}$ = 28.35 mol m^{-2} / (5 m – 0 m)

$C_{Tot\ rain\ NO3}$ = 5.67 mol m^{-3}

Residual (urine) component. With all of the known components accounted for, we calculate residual [NO_3^-] ($C_{residual\ NO3}$) found in the Level 2 archaeological midden samples by subtracting each previously described contributor from the total [NO_3^-] ($C_{Tot\ NO3}$)

9) $C_{residual\ NO3} = C_{Tot\ NO3} - C_{Tot\ ash\ NO3} - C_{Tot\ cd\ NO3} - C_{Tot\ rain\ NO3}$

E) $C_{residual\ NO3}$ = 43.22 mol m^{-3} – 1.84 mol m^{-3} – 0.090 mol m^{-3} – 5.67 mol m^{-3}

$C_{residual\ NO3}$ = 35.62 mol m^{-3}

C) Modeling Organism Populations

Organism density ($D_{org\ i}$). We interpret $C_{residual\ NO3}$ *to* reflect NO_3^- introduced into Level 2 by human and caprine urination. To calculate the density of organisms required to produce $C_{residual\ NO3}$ we first need to estimate the rate that the average yearly addition of NO_3^- by organisms per square meter based on modern data available for urination rates (U_R) and [NO_3^-] in urine ($C_{urine\ NO3}$), the latter corrected for ammonia volatilization (F_{av}) (see tables S3-S5). To calculate organism density ($D_{org\ NO3}$, in organisms m^{-2}), we take the product of $C_{urine\ NO3}$ times the urination rate per year (U_R), times one minus the runoff ratio (α), where α = 0, and so 1 – α = 1, multiplied by the fraction of time spent on the tell by the organisms (F_{om}). This is then divided by the sedimentation rate, and finally the residual component is divided by this final value

10) $D_{org\ NO3} = C_{residual\ NO3} / ((C_{urine\ NO3} * U_R * (1 - \alpha) * F_{om}) / \Gamma)$

F) $D_{org\ NO3}$ = 35.62 mol m^{-3} / (6.21E-02 mol L^{-1} org^{-1} * 4.11E+02 L yr^{-1} * (1 – 0) * 0.5) / 0.0165 m yr^{-1})

$D_{org\ NO3}$ = 3.7E-02 org m^{-2}

This final value is the density of organisms ($D_{org\ NO3}$) required to produce $C_{residual\ NO3}$ in Level 2 midden. To calculate the average organismal population ($N_{org\ NO3}$) at any given moment for Level 2, we multiplied the organism density, $D_{org\ NO3}$, by the estimated area of level 2 (A), which in this case is considered the area (57,227 m^2) of the base of the tell

11) $N_{org\ NO3} = D_{org\ NO3} * A$

G) $N_{org\ NO3}$ = 3.7E-02 org m^{-2} * 57,227 m^2

$N_{org\ NO3}$ = 2096 organisms

The example from Level 2 used here is based on a constant sedimentation rate throughout the accumulation period of the tell. Population densities derived using a variable sedimentation rate are shown in Fig. 6 (see supplemental section 9 for details on sedimentation rates). In addition, average $C_{residual\ i}$ for the entire mound (taking the unweighted values from all levels), and their associated $D_{org\ i}$ values are calculated following the same procedure described here.

Section S9. Calculation of sedimentation rates

The estimate of the $D_{org\ i}$ required to produce [Na^+], [Cl^-], and [NO_3^-] found in each archaeological level at Aşıklı Höyük is computed in two steps. The first follows the mass balance calculation to find $C_{residual\ i}$. The second quantifies the amount of each soluble salt constituent produced by a single organism in a single vertical meter, which requires a sedimentation rate. To remain conservative in our population estimates, we use two different methods in determining our sedimentation rate based on work by Quade et al., (2018) (*8*). The first is to use dates of the top and bottom of the mound and assume a constant sedimentation rate for its entirety. An average of ~0.0165 m yr^{-1} is derived from this procedure, and this has been applied to Levels 5-2 (Fig. 6a). The second, "variable sedimentation rate" method determines individual sedimentation rates for each archaeological level based on carbon-14 ages for the boundaries between them. The average ages and approximate thicknesses of the four levels in question are found in (*8*), and the corresponding sedimentation rates listed in tables S3-S5 (see Fig. 6b for final population densities based on variable sedimentation rates). Due to the lack of reliable dates in Level 5 at the time of publication, the constant sedimentation rate of 0.0165 m yr^{-1} from the first calculation

is applied to Level 5 in the second. Arguments for and against each of these dating schemes are described in (*8*).

Section S10. Heterogeneity of elemental concentrations across various samples

The architectural and other human-built features display spatial heterogeneity in each level of Aşıklı Höyük (*6, 56*). However, the sediments and refuse in all levels tend to be admixed on account of frequent clean-up of activity areas by the inhabitants, and periodic leveling and in-filling of pits and buildings. Urine inputs may or may not be as admixed as bone and artifact debris, but sediments were also moved around frequently in connection with rebuilding, resurfacing, and leveling of indoor and outdoor areas. Thus, it is reasonable to expect some degree of spatial averaging of urine-derived chemical traces in the sediment samples by period. This also can explain the higher soluble salt concentrations in found in some of the brick, floor, plaster, or hearth samples in later levels. If there is usage of midden to form the structural components of the architecture, as well as infilling of old building with midden (and its potential subsequent use as a corralling area), then this is likely to impart some high salt concentration on these supposedly “urine-free” samples.

Section S11. Sensitivity of the mass balance model

Aside from analytical errors listed in table S1, no estimates of fully propagated error are provided in the main text, because we believe this fails to express the full uncertainties. The largest sources of uncertainty in our reconstruction of organism densities and numbers using [Na^+], [Cl^-] and [NO_3^-] are their representative values in wood ash, rainfall during the Holocene, the runoff ratio, and the fraction of time on and off the mound by animals and inhabitants. For

$C_{residual\ Na}$ specifically, the biggest uncertainty is the % wood ash in the mound, and its average [Na+]. For [$NO3^-$], the largest uncertainty is the fraction volatilized. Of the three elements, estimation of [Cl^-] is the least uncertain.

Our approach to the error issue here is to describe the factor increases/decreases in each constituent (and their various components) required to completely account for $C_{residual\ i}$, and to derive $D_{org\ i}$. All values presented after this point are based on the average values from all midden samples. For [Na^+], less than a doubling of wood ash concentrations ($C_{ash\ Na}$) and wood ash density are needed to completely explain soluble [Na^+] anomalies. However, although there is a large range in [Na^+] between tree species, and the components of the tree that is burned, we find our values to be representative of an average of relevant species for burned inner wood and bark (*26, 67*). For a given species, it has been shown that [Na^+] only varies by a factor of ~2 based on inner wood and bark ash residue (*26*). As such, our use of an average value for $C_{ash\ Na}$, which falls in the middle of the factor-of-two range, implies that a doubling of [Na^+] in wood ash is unlikely. Any [Na^+] lower than our stated value would lead to higher $C_{residual\ Na}$, and an increase in estimated $D_{org\ Na}$, which is likely based on the work of (*67*). Our future work involves expanding the measurements of burned ash from tree species near the archaeological site to better constrain our estimates.

In addition, [Na^+] in rain and rainfall amount per year must be a factor of ~10x higher, or a ~10x reduction in runoff ratio is needed to account for soluble [Na^+] in the mound. There is no evidence from a variety of records for large long-term variations in rainfall in Turkey over the Holocene (*64-66*), meaning 400 mm yr^{-1} is a suitable value. It is likely that the runoff ratio varied, especially on the mound periphery compared to the shallower dipping top, but many samples are from the steeper sides of the tell, implying high runoff ratios. Supplement section #5

provides and overview of this issue. Concentration of the salts of interest in rain can vary, as it is dependent on a host a factors including average storm trajectories, and these may have shifted during the Holocene.

With all factors taken into account that affect the mass-balance portion of our model, our estimates of salt residuals for [Na^+], [Cl^-] and [NO_3^-], and their subsequent population estimates are very likely lower limits. In terms of uncertainties of our population density estimates using the residuals, two variables are likely to have the largest impact on uncertainty. For the fraction of time spent on the mound, it is likely that our value of 0.5 (or half of the day) is too low, for socioeconomic reasons present in the archaeological background portion of the main text, and supplement section #6. Increasing this value would then decrease our estimates of organisms. The fraction of N from urine that is volatilization represents the largest uncertainty for nitrate-based organism estimates. While we adopt a value of 45% based on climatic conditions of central Turkey, this value has been shown to range from <5% to >60% (*46-50*). Estimates higher than our value will lead to increased $D_{org\ i}$ and $N_{org\ i}$, while lower values will decrease our model results for $D_{org\ i}$ and $N_{org\ i}$.

We have already provided some general reasoning behind excluding dung, but acknowledge that dung likely did add some amount of soluble salts to the tell during occupation. Inclusion of dung would also drive our estimates down, but as with all other estimates that may be described as maximums, they would not affect the relative changes between levels.

Supplementary Figures

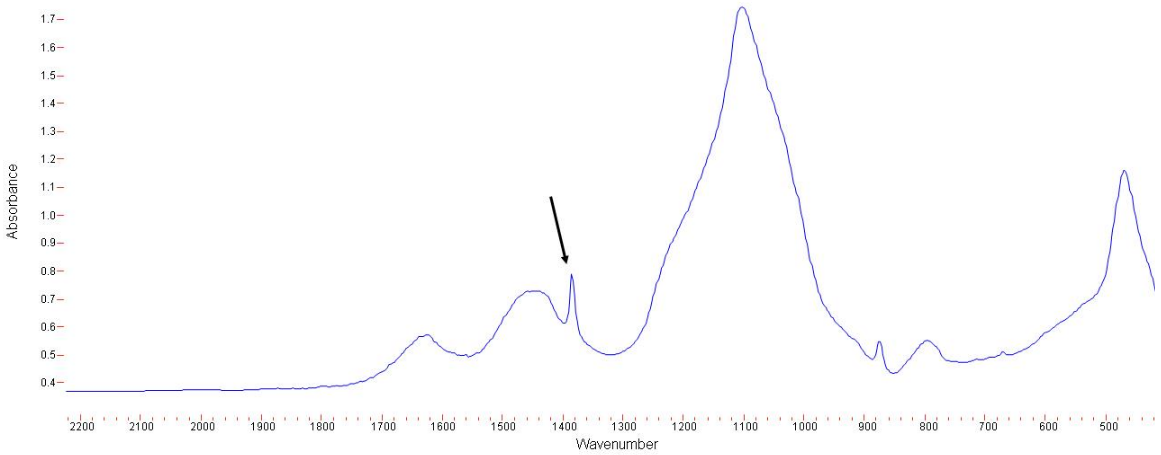

Fig. S1. Infrared spectrum from a dung layer in a midden (level 3). The strong peak at 1385 cm^{-1} (arrow), indicates the presence of nitratine. Sample prepared using a KBr pellet and spectrum collected at 4 cm^{-1} resolution, 32 co-added scans.

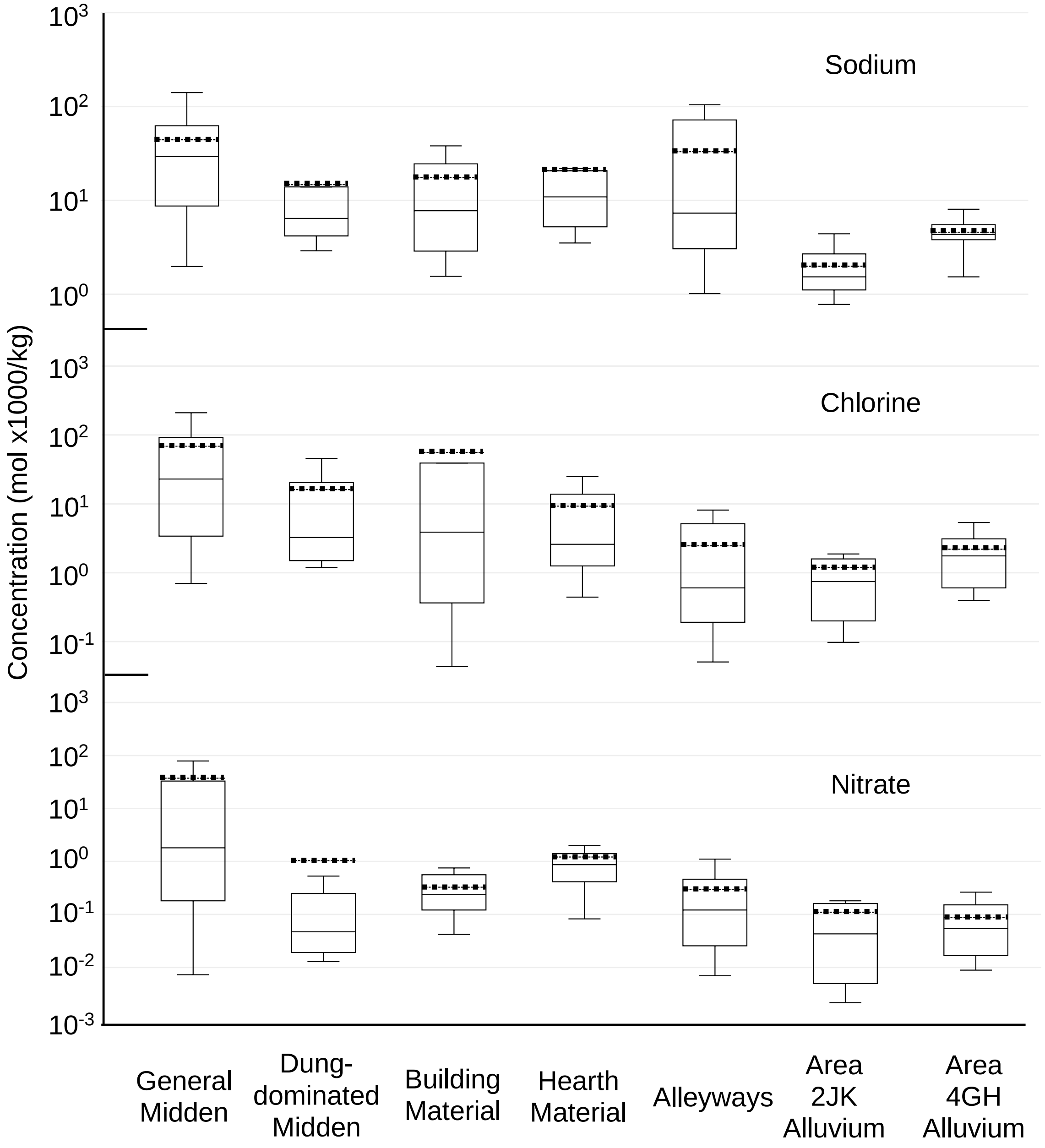

Fig. S2. Comparison of salt concentrations in various archaeological and nonarchaeological materials. Box and whisker plot of soluble sodium (Top), chlorine (Middle), and nitrate (Bottom) concentrations (in moles x1000/kg) for various archaeological and non-archaeological materials. Whisker lengths are based on 1/3 of the interquartile range. The solid line within each box represents the median, while the dotted line shows the mean of the sample set.

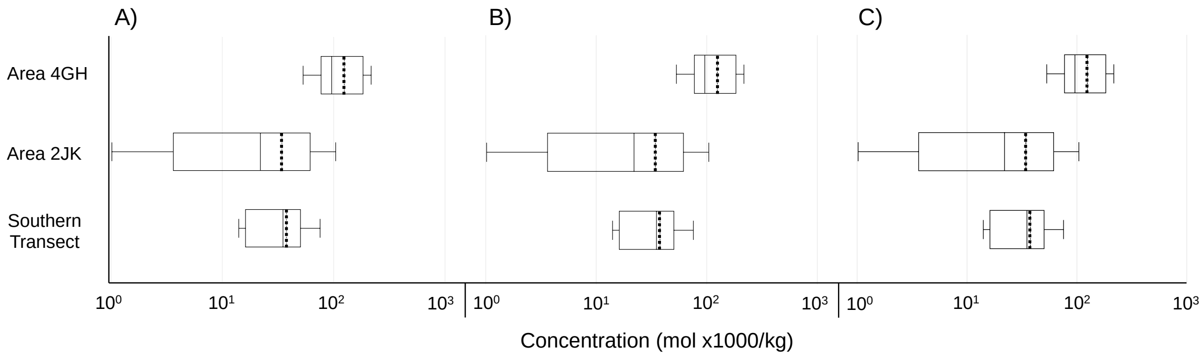

Fig. S3. Box and whisker plot of soluble salt concentrations (in moles × 1000 kg^{-1}) across three sampling sections: area 4GH, area 2JK, and southern transect. A) [Na^+], **B)** [Cl^-] , and **C)** [NO_3^-]. Whisker lengths are based on 1/3 of the interquartile range. The solid line within each box represents the median, while the dotted line shows the mean of the sample set. To provide consistency, only general midden, dung-dominated midden, and alleyway samples from Level 2 are considered here.

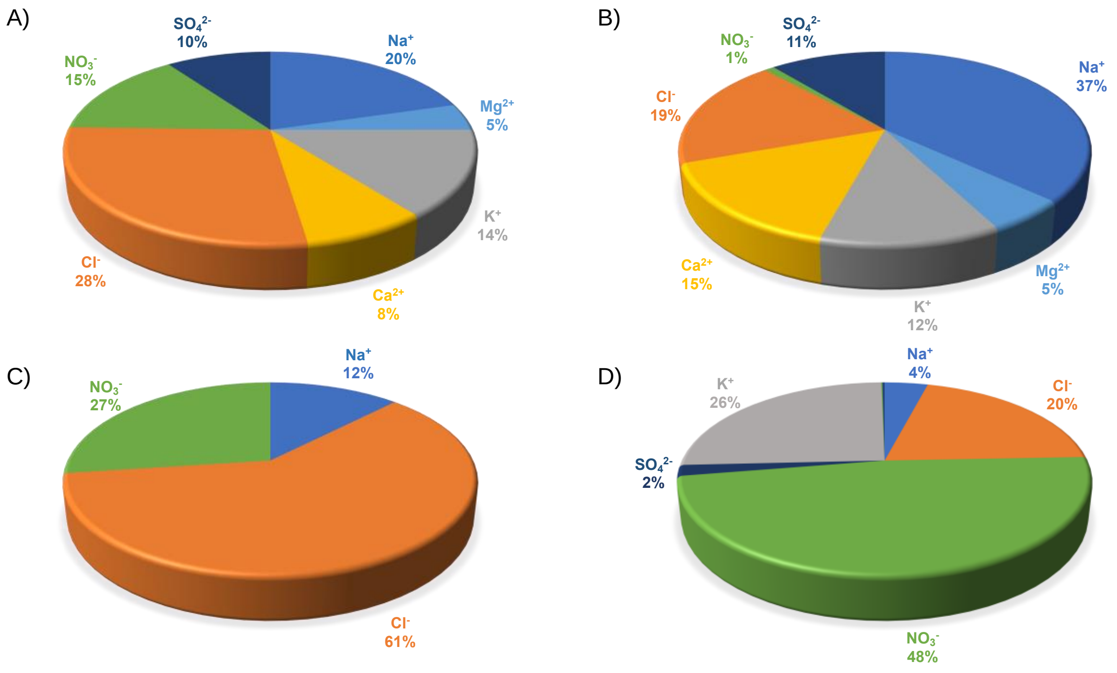

Fig. S4. Four pie diagrams displaying soluble salt percentages. A) Total soluble salts of all midden samples from Aşıklı Höyük, **B)** Total soluble salts of all underlying natural alluvium from Aşıklı Höyük, **C)** Leftover salts after mass balance model is applied, and **D)** Modern urine composition based on averages from humans and sheep.

Table S1. Soluble salt chemistry and $\delta^{15}N_{soluble}$ of archaeological and nonarchaeological layers at Aşıklı Höyük.

Sample ID	Asikli ID	Sample Type	Level	[Na]* [mol/kg]	[Mg]* [mol/kg]	[K]* [mol/kg]	[Ca]* [mol/kg]	[Cl]* [mol/kg]	[NO3]* [mol/kg]	[SO4]* [mol/kg]	δ15N † per mil
AHJQ-790	1148	Charcoal and Hearth Material	2	1.007E-02	3.140E-03	1.903E-02	2.717E-02	1.118E-03	4.069E-04	4.631E-02	12.0
AHJQ-791	1149	Charcoal and Hearth Material	2	1.669E-02	2.864E-03	1.087E-02	1.309E-02	1.679E-03	4.342E-04	2.847E-02	5.5
AHJQ-792	1150	Charcoal and Hearth Material	2	8.802E-02	5.340E-03	2.842E-02	1.726E-02	4.716E-03	1.288E-03	9.155E-02	8.5
AHJQ-793	1151	Charcoal and Hearth Material	2	3.680E-03	3.831E-04	7.748E-03	1.280E-03	5.342E-04	2.785E-04	2.444E-03	6.6
AHJQ-794	1152	Charcoal and Hearth Material	2	3.520E-03	3.463E-03	1.309E-02	2.419E-02	4.441E-04	8.237E-05	3.522E-02	No Signal
AHJQ-784	1142	Floor	2	2.629E-03	2.558E-04	3.640E-03	3.043E-04	4.367E-05		1.563E-03	No Signal
AHJQ-785	1143	Floor	2	1.570E-02	1.381E-03	1.230E-02	2.177E-03	2.548E-03	7.541E-04	1.426E-02	11.2
AHJQ-786	1144	Floor	2	1.555E-03	2.662E-04	3.951E-03	3.873E-04	5.961E-05	4.200E-05	6.687E-04	No Signal
AHJQ-778	1136	Alleyway	2	1.776E-03	2.414E-04	6.522E-03	2.231E-03	5.073E-05	6.788E-05	4.165E-03	No Signal
AHJQ-779	1137	Alleyway	2	1.019E-03	3.213E-04	4.868E-03	1.051E-03	5.591E-05	6.947E-06	1.323E-03	14.9
AHJQ-780	1138	Alleyway	2	7.332E-03	2.455E-04	6.826E-03	1.009E-03	3.188E-04		4.056E-03	11.1
AHJQ-781	1139	Alleyway	2	2.197E-02	4.963E-03	1.566E-02	1.368E-02	1.289E-03	1.160E-05	3.865E-02	No Signal
AHJQ-782	1140	Alleyway	2	3.477E-03	6.976E-04	9.193E-03	5.506E-03	2.352E-04	3.966E-05	1.090E-02	13.0
AHJQ-783	1141	Alleyway	2	4.082E-03	1.261E-03	1.088E-02	3.410E-03	6.043E-04	1.743E-04	1.160E-02	10.5
AHJQ-787	1145	Alleyway	2	1.043E-01	8.377E-03	2.483E-02	6.100E-03	8.129E-03	1.110E-03	9.082E-02	17.2
AHJQ-788	1146	Alleyway	2	6.681E-02	9.254E-03	1.976E-02	1.677E-02	4.593E-03	3.992E-04	7.988E-02	9.1
AHJQ-789	1147	Alleyway	2	8.687E-02	1.708E-02	2.551E-02	3.531E-02	6.834E-03	5.276E-04	1.448E-01	11.0
AHJQ-766	1112	Bulk Midden	2	3.439E-02	9.340E-03	3.857E-02	2.847E-02	1.167E-01	2.375E-02	1.026E-02	16.0
AHJQ-766	1113	Bulk Midden	2	4.537E-02	7.713E-03	3.368E-02	2.309E-02	9.681E-02	2.606E-02	1.300E-02	16.5
AHJQ-813	1174	Bulk Midden	2	5.314E-02	1.522E-03	1.264E-02	3.117E-03	4.937E-02	6.720E-04	1.105E-02	6.1
AHJQ-813	1175	Bulk Midden	2	8.514E-02	5.536E-03	2.287E-02	8.642E-03	1.205E-01	9.174E-04	1.724E-02	6.0
AHJQ-813	1176	Bulk Midden	2	9.596E-02	3.243E-03	2.699E-02	7.203E-03	7.276E-02	7.317E-04	4.553E-02	8.9
AHJQ-814	1177	Bulk Midden	2	2.171E-01	2.777E-02	5.305E-02	2.006E-02	3.319E-01	7.672E-03	4.698E-02	5.8
AHJQ-815	1178	Bulk Midden	2	1.723E-01	3.611E-02	5.647E-02	8.077E-03	1.951E-01	2.221E-04	7.908E-02	No Signal
AHJQ-21		Bulk Midden	2	4.402E-02	1.941E-02	7.449E-02	3.810E-02	5.738E-02	1.386E-01	2.994E-02	13.7
AHJQ-2J		Bulk Midden	2	7.558E-02	1.771E-02	7.490E-02	2.738E-02	4.668E-02	1.104E-01	2.050E-02	13.7
AHJQ-19		Bulk Midden	2	5.033E-02	3.693E-02	7.849E-02	6.371E-02	7.650E-02	2.073E-01	3.079E-02	13.8
AHJQ-18		Bulk Midden	2	2.616E-02	7.806E-03	5.253E-02	1.401E-02	2.305E-02	6.352E-02	1.477E-02	14.0
AHJQ-17		Bulk Midden	2	1.615E-02	5.532E-03	3.625E-02	8.020E-03	1.557E-02	4.084E-02	8.441E-03	14.3
AHJQ-16		Bulk Midden	2	1.405E-02	3.023E-03	3.089E-02	5.394E-03	1.027E-02	2.533E-02	7.492E-03	14.3
AHJQ-799	1157	Bulk Hearth	3	4.882E-02	3.928E-03	1.018E-02	7.478E-03	3.839E-02	4.940E-03	1.736E-02	8.5
AHJQ-795	1153	Charcoal and Hearth Material	3	3.970E-03	1.178E-03	5.215E-03	3.693E-03	2.593E-03	8.657E-04	1.722E-03	No Signal
AHJQ-796	1154	Charcoal and Hearth Material	3	1.089E-02	1.389E-03	1.411E-02	4.373E-03	2.489E-02	1.994E-03	3.649E-03	No Signal
AHJQ-762	1105	Bulk Dung	3	8.886E-03	1.505E-03	5.863E-03	3.790E-03	1.194E-02	1.945E-05	1.213E-03	No Signal
AHJQ-763	1106	Bulk Dung	3	6.587E-02	2.705E-03	2.317E-02	5.865E-03	7.143E-02	5.262E-04	1.404E-02	9.5
AHJQ-825	1293	Bulk Dung	3	6.512E-03	8.578E-04	8.611E-03	2.847E-03	2.986E-03	1.543E-04	1.353E-03	No Signal
AHJQ-826	1294	Bulk Dung	3	2.902E-02	1.520E-03	2.571E-02	1.132E-02	4.568E-02	8.503E-03	1.109E-02	15.9
AHJQ-825	1292	Phytoliths mixed with Dung	3	4.690E-03	6.229E-04	7.332E-03	1.455E-03	1.311E-03	4.692E-05	4.752E-04	No Signal
AHJQ-758	1100	Brick	3	1.990E-02	3.686E-04	4.086E-03	1.319E-03	8.771E-03	2.354E-04	3.408E-03	8.1
AHJQ-758	1098	Bulk Midden	3	1.318E-02	6.596E-04	5.132E-03	2.104E-03	3.673E-03	4.104E-05	3.293E-03	13.3
AHJQ-758	1099	Bulk Midden	3	1.869E-02	7.327E-04	5.342E-03	1.291E-03	6.104E-03	7.266E-06	4.910E-03	13.4
AHJQ-758	1101	Bulk Midden	3	3.047E-02	4.152E-04	5.209E-03	1.951E-03	1.815E-02	7.460E-04	4.309E-03	10.5
AHJQ-764	1107	Bulk Midden	3	1.217E-01	1.688E-02	2.933E-02	2.004E-02	2.448E-01	4.367E-03	7.903E-03	8.2
AHJQ-765	1108	Bulk Midden	3	2.929E-02	2.242E-03	1.209E-02	3.612E-03	2.336E-02	7.466E-04	1.022E-02	9.5
AHJQ-766	1109	Bulk Midden	3	4.352E-03	6.499E-04	8.807E-03	2.769E-03	4.609E-03	4.864E-04	2.039E-03	15.5
AHJQ-766	1110	Bulk Midden	3	1.822E-02	3.768E-03	3.017E-02	1.170E-02	7.719E-02	1.876E-03	2.653E-04	14.9
AHJQ-766	1111	Bulk Midden	3	4.621E-02	3.397E-02	5.851E-02	8.364E-02	3.466E-01	2.480E-02	8.947E-04	17.3
AHJQ-767	1114	Bulk Midden	3	7.877E-03	9.431E-04	1.134E-02	2.075E-03	3.304E-03	1.801E-04	7.398E-03	15.3
AHJQ-767	1115	Bulk Midden	3	8.236E-02	2.951E-02	7.181E-02	9.812E-02	3.674E-01	5.101E-02	5.159E-02	16.0
AHJQ-767	1116	Bulk Midden	3	6.261E-02	2.871E-02	3.445E-02	5.494E-02	2.251E-01	3.281E-02	4.410E-02	16.7
AHJQ-767	1117	Bulk Midden	3	4.519E-02	1.798E-02	4.210E-02	3.878E-02	1.329E-01	2.231E-02	5.025E-02	16.3
AHJQ-767	1118	Bulk Midden	3	3.007E-02	6.496E-03	2.230E-02	1.764E-02	2.292E-02	4.601E-03	4.029E-02	17.3
AHJQ-767	1119	Bulk Midden	3	7.299E-02	9.789E-03	2.998E-02	1.921E-02	8.657E-03	1.729E-03	7.610E-02	15.6
AHJQ-3		Bulk Midden	3	2.330E-02	3.122E-03	3.610E-02	6.400E-03	1.781E-02	2.939E-02	1.251E-02	13.5
AHJQ-15		Bulk Midden	3	1.624E-01	6.128E-02	2.587E-01	9.852E-02	1.759E-01	4.542E-01	8.254E-02	13.6
AHJQ-14		Bulk Midden	3	6.189E-02	1.973E-02	7.334E-02	2.947E-02	6.438E-02	1.197E-01	2.964E-02	13.5
AHJQ-3A1		Bulk Midden	3	3.425E-02	3.858E-03	4.436E-02	7.455E-03	2.367E-02	3.524E-02	1.176E-02	13.0
AHJQ-3B		Bulk Midden	3	6.857E-02	1.004E-02	5.318E-02	1.410E-02	6.944E-02	3.656E-02	8.015E-03	13.8
AHJQ-3D		Bulk Midden	3	1.410E-01	7.441E-02	1.081E-01	7.912E-02	2.096E-01	3.026E-01	3.575E-02	14.1
AHJQ-3E		Bulk Midden	3	5.851E-02	1.186E-02	6.234E-02	2.265E-02	5.470E-02	7.875E-02	3.149E-02	14.1
AHJQ-798	1156-a	Bulk Hearth	4	9.022E-03	3.932E-04	5.039E-03	1.956E-03	1.827E-03	5.576E-04	1.286E-03	8.2
AHJQ-798	1156-b	Bulk Hearth	4	2.190E-02	3.009E-04	8.324E-03	1.029E-03	1.086E-02	1.029E-03	3.222E-03	8.5
AHJQ-775	1127	Bulk Midden	4	5.885E-03	3.278E-04	3.699E-03	1.098E-03	6.999E-04	4.808E-05	4.598E-04	No Signal
AHJQ-775	1128	Bulk Midden	4	1.192E-02	5.076E-04	4.942E-03	1.807E-03	2.127E-03	9.414E-05	8.448E-04	13.5
AHJQ-775	1129	Bulk Midden	4	1.120E-02	6.366E-04	4.503E-03	3.171E-03	1.209E-03	5.713E-05	3.735E-04	10.0
AHJQ-775	1130	Bulk Midden	4	1.929E-02	1.027E-03	7.977E-03	5.119E-03	8.169E-03	6.281E-04	7.303E-04	11.1
AHJQ-775	1131	Bulk Midden	4	5.139E-03	5.214E-04	7.453E-03	1.907E-03	1.412E-03	5.612E-05	3.542E-04	17.7
AHJQ-775	1132	Bulk Midden	4	1.164E-02	4.343E-04	1.125E-02	1.782E-03	1.413E-02	6.016E-04	4.156E-04	16.2
AHJQ-775	1133	Bulk Midden	4	7.691E-03	5.363E-04	1.129E-02	2.259E-03	1.499E-02	2.093E-04	9.145E-04	No Signal
AHJQ-497		Bulk Midden	4	2.928E-02	1.571E-03	8.700E-03	4.539E-03	1.752E-02	3.920E-03	9.836E-03	No Signal
AHJQ-494		Bulk Midden	4	6.740E-02	1.159E-02	6.995E-03	3.946E-02	1.162E-01	9.143E-03	1.301E-02	No Signal
AHJQ-797	1155	Bulk Hearth	5	1.226E-02	2.109E-03	6.043E-03	6.438E-03	1.481E-02	1.447E-03	8.447E-03	7.7
AHJQ-823	1288	Bulk Dung	5	4.461E-03	1.534E-03	4.107E-03	3.833E-03	5.341E-03	1.204E-04	9.533E-04	No Signal
AHJQ-823	1289	Bulk Dung	5	6.420E-03	1.002E-03	5.206E-03	3.781E-03	3.245E-03	2.716E-05	8.422E-04	No Signal
AHJQ-824	1290	Bulk Dung	5	3.321E-03	1.112E-03	4.315E-03	2.643E-03	1.193E-03	1.831E-05	7.255E-04	17.1
AHJQ-824	1291	Bulk Dung	5	2.911E-03	8.472E-04	4.381E-03	2.688E-03	1.559E-03	1.285E-05	1.006E-03	No Signal
AHJQ-810	1171	Brick	5	6.155E-02	2.897E-02	1.786E-02	7.208E-02	3.503E-01	1.455E-04	8.561E-03	No Signal
AHJQ-811	1172	Brick	5	3.820E-02	9.137E-03	1.264E-02	2.810E-02	1.304E-01	1.133E-04	9.050E-03	No Signal
AHJQ-812	1173	Brick	5	2.963E-03	3.151E-04	2.058E-03	9.590E-04	4.666E-04		2.602E-04	No Signal
AHJQ-758	1096	Plaster	5	7.756E-03	4.999E-04	3.684E-03	1.408E-03	5.323E-03	6.291E-04	2.215E-03	7.0
AHJQ-758	1097	Plaster	5	7.276E-03	5.714E-04	3.403E-03	1.448E-03	3.870E-03	3.602E-04	1.630E-03	9.4
AHJQ-751	1084	Bulk Midden	5	3.375E-03	7.159E-04	2.513E-03	2.955E-03	1.010E-03	3.066E-05	9.260E-04	No Signal
AHJQ-751	1085	Bulk Midden	5	3.134E-03	6.211E-04	2.729E-03	2.696E-03	9.892E-04	9.986E-05	9.356E-04	No Signal
AHJQ-751	1086	Bulk Midden	5	4.220E-03	7.591E-04	4.195E-03	3.773E-03	7.937E-04	8.987E-05	1.286E-03	No Signal
AHJQ-751	1087	Bulk Midden	5	3.373E-03	6.547E-04	3.475E-03	2.069E-03	1.199E-03	1.273E-04	9.081E-04	No Signal
AHJQ-809	1167	Bulk Midden	5	1.981E-03	4.646E-04	1.621E-03	1.765E-03	7.170E-04		2.309E-04	13.0
AHJQ-809	1168	Bulk Midden	5	2.813E-03	5.025E-04	3.413E-03	2.325E-03	7.035E-04	3.369E-05	2.607E-04	13.5
AHJQ-809	1169	Bulk Midden	5	6.587E-03	3.391E-04	5.063E-03	2.188E-03	2.486E-03	2.329E-04	7.214E-04	8.1
AHJQ-809	1170	Bulk Midden	5	4.664E-03	3.294E-04	4.609E-03	1.805E-03	2.043E-03	3.188E-05	2.095E-04	No Signal
AHJQ-818 (IA)	non-archeological	Bulk Sediment	6	1.532E-03	1.349E-04	7.336E-04	2.458E-04	6.831E-04	5.773E-05	3.422E-04	No Signal
AHJQ-818 (IIA-IIB)	non-archeological	Bulk Sediment	6	4.643E-03	1.095E-03	1.781E-03	3.886E-03	7.022E-03	1.124E-04	3.349E-03	No Signal
AHJQ-818 (IIC)	non-archeological	Bulk Sediment	6	4.208E-03	3.461E-04	1.107E-03	5.808E-04	5.810E-04	8.875E-06	7.056E-04	No Signal
AHJQ-818-2 (IIIB)	non-archeological	Bulk Sediment	6	3.224E-03	5.659E-04	1.138E-03	3.133E-03	3.066E-03	2.326E-04	1.237E-03	17.4
AHJQ-820 (IIIB-Char.)	non-archeological	Bulk Sediment	6	4.338E-03	3.911E-04	1.539E-03	1.329E-03	3.956E-04	1.797E-04	5.902E-04	No Signal
ASI-15 JQ-08		Bulk Sediment	6	8.061E-03	3.125E-04	1.158E-03	1.562E-03	1.571E-03	2.005E-05	9.879E-04	No Signal
ASI-15 JQ-07		Bulk Sediment	6	5.558E-03	1.015E-03	1.646E-03	2.008E-03	6.098E-04	1.694E-05	7.288E-04	No Signal
ASI-15 JQ-06		Bulk Sediment	6	4.002E-03	1.984E-03	1.289E-03	1.283E-03	4.239E-04	1.294E-05	6.275E-04	No Signal
ASI-15 JQ-04		Bulk Sediment	6	5.499E-03	5.093E-04	1.018E-03	1.822E-03	1.758E-03	1.647E-05	2.281E-03	No Signal
ASI-15 JQ-01		Bulk Sediment	6	5.476E-03	9.484E-04	1.048E-03	4.318E-03	5.363E-03	1.794E-05	2.631E-03	No Signal
AHJQ-751	1080-1	Bulk Soil	6	7.785E-04	1.412E-04	3.434E-04	2.556E-04	1.493E-04	2.160E-06	7.880E-05	No Signal
AHJQ-751	1080-2	Bulk Soil	6	1.184E-03	2.245E-04	3.799E-04	7.890E-04	2.495E-04		8.234E-05	No Signal
AHJQ-751	1081	Bulk Soil	6	1.034E-03	1.794E-04	4.900E-04	4.397E-04	9.767E-05	4.570E-06	4.320E-05	No Signal
AHJQ-751	1082	Bulk Soil	6	1.366E-03	2.108E-04	1.033E-03	6.872E-04	5.444E-04	4.284E-05	1.202E-04	No Signal
AHJQ-751	1083	Bulk Soil	6	1.704E-03	3.075E-04	1.211E-03	7.918E-04	1.301E-03	9.929E-05	4.746E-04	No Signal
AHJQ-758	1094	Bulk Soil	6	2.150E-03	2.250E-04	7.423E-04	2.358E-04	9.476E-04	6.001E-06	4.548E-04	No Signal
AHJQ-758	1095-1	Bulk Soil	6	4.405E-03	5.770E-04	1.926E-03	1.123E-03	4.355E-03	4.352E-04	9.285E-04	No Signal
AHJQ-758	1095-2	Bulk Soil	6	3.242E-03	2.843E-04	1.685E-03	5.663E-04	1.877E-03	1.806E-04	5.789E-04	No Signal
AHJQ-821 (IIA-IIB)	non-archeological	Carbonate Nodule	6	2.522E-03	3.715E-04	7.031E-04	1.759E-03	1.841E-03	1.408E-04	9.136E-04	No Signal
AHJQ-818 (IB)	non-archeological	Carbonate Nodule + Sediment	6	4.271E-03	2.373E-04	1.769E-03	6.045E-04	2.016E-03	2.637E-04	1.124E-03	No Signal
AHJQ-819b (IIC)	non-archeological	Organic Matter in Clay Medium	6	6.287E-03	1.019E-03	1.741E-03	3.721E-03	3.213E-03	5.445E-05	4.113E-03	No Signal
AHJQ-828	non-archeological	Holocene Sediment	Out of Context	3.637E-04	8.876E-04	2.678E-03	2.497E-03	6.966E-05	9.753E-06	7.349E-05	6.0
AHJQ-816	non-archeological	Modern Alluvium	Out of Context	7.641E-02	1.070E-02	1.984E-02	8.346E-03	7.620E-02	4.443E-02	9.146E-03	7.8
AHJQ-829	non-archeological	Pleistocene Sediment	Out of Context	1.193E-02	3.756E-04	8.949E-04	2.648E-03	8.301E-03	2.117E-03	1.284E-03	2.3

*All concentrations have a maximum of 5% analytical error

† Conservative error of 0.62 per mil for each sample based on analytical error

Table S2. Statistical information of soluble salts based on material, spatial, and temporal setting.

Group 1: Material Type (mol/kg)	General Midden	Dung-Dominated Midden	Construction Material	Hearths	Alleyways	Natural Alluvium (Area 2JK)	Natural Alluvium (Area 4GH)
Sodium (mol/kg)							
Number	51	9	9	11	9	8	13
Mean	4.44E-02	1.47E-02	1.75E-02	2.08E-02	3.31E-02	1.98E-03	4.59E-03
Median	2.93E-02	6.42E-03	7.76E-03	1.09E-02	7.33E-03	1.54E-03	4.34E-03
Standard Deviation (1σ)	4.80E-02	2.08E-02	2.02E-02	2.57E-02	4.12E-02	1.25E-03	1.67E-03
Percent Standard Deviation (1σ)	108.2	141.8	115.4	123.7	124.7	63.0	36.5
Chlorine (mol/kg)							
Number	51	9	9	11	9	8	13
Mean	6.86E-02	1.61E-02	5.58E-02	9.26E-03	2.46E-03	1.19E-03	2.20E-03
Median	2.29E-02	3.25E-03	3.87E-03	2.59E-03	6.04E-04	7.46E-04	1.76E-03
Standard Deviation	9.58E-02	2.51E-02	1.18E-01	1.23E-02	3.20E-03	1.42E-03	2.03E-03
Percent Standard Deviation (1σ)	139.6	156.4	212.1	133.3	130.1	119.4	92.6
Nitrate (mol/kg)							
Number	50	9	7	11	8	7	13
Mean	3.73E-02	1.05E-03	3.26E-04	1.21E-03	2.92E-04	1.10E-04	8.73E-05
Median	1.80E-03	4.69E-05	2.35E-04	8.66E-04	1.21E-04	4.28E-05	5.45E-05
Standard Deviation	8.32E-02	2.80E-03	2.72E-04	8.66E-04	3.82E-04	1.58E-04	9.00E-05
Percent Standard Deviation (1σ)	223.3	267.3	83.4	71.5	130.9	143.1	103.1

Group 2: Spatial Distribution (general dung, dung-dominated, and alleyway midden in Level 2) (mol/kg)	Area 4GH	Area 2JK	Southern Transect
Sodium (mol/kg)			
Number	6	11	5
Mean	3.77E-02	3.43E-02	1.25E-01
Median	3.51E-02	2.20E-02	9.60E-02
Standard Deviation	2.36E-02	3.71E-02	6.77E-02
Percent Standard Deviation (1σ)	62.6	108.1	54.2
Chlorine (mol/kg)			
Number	6	11	5
Mean	3.82E-02	2.14E-02	1.54E-01
Median	3.49E-02	1.29E-03	1.21E-01
Standard Deviation	2.62E-02	4.25E-02	1.14E-01
Percent Standard Deviation (1σ)	68.5	198.5	74.1
Nitrate (mol/kg)			
Number	6	10	5
Mean	9.77E-02	5.22E-03	2.04E-03
Median	8.69E-02	2.87E-04	7.32E-04
Standard Deviation	6.85E-02	1.04E-02	3.16E-03
Percent Standard Deviation (1σ)	70.2	199.4	154.5

Group 3: Temporal Variability (mol/kg)	Level 5	Level 4	Level 3	Level 2	Sterile Material (Area 4GH and Area 2JK)	Sterile Material (Area 2JK only)
Sodium (mol/kg)						
Number	12	9	26	22	21	8
Mean	3.94E-03	1.88E-02	4.80E-02	5.58E-02	3.59E-03	1.98E-03
Median	3.37E-03	1.16E-02	3.24E-02	4.47E-02	4.00E-03	1.54E-03
Standard Deviation	1.41E-03	1.97E-02	4.19E-02	5.59E-02	1.98E-03	1.25E-03
Percent Standard Deviation (1σ)	35.8	104.7	87.4	100.2	55.0	63.0
Chlorine (mol/kg)						
Number	12	9	26	22	21	8
Mean	1.77E-03	1.96E-02	8.59E-02	5.61E-02	1.81E-03	1.19E-03
Median	1.20E-03	8.17E-03	3.47E-02	1.93E-02	1.30E-03	7.46E-04
Standard Deviation	1.37E-03	3.68E-02	1.08E-01	8.06E-02	1.85E-03	1.42E-03
Percent Standard Deviation (1σ)	77.2	187.8	126.2	143.7	102.3	119.4
Nitrate (mol/kg)						
Number	11	9	26	21	20	7
Mean	7.50E-05	1.64E-03	4.66E-02	3.09E-02	9.53E-05	1.10E-04
Median	3.37E-05	2.09E-04	4.48E-03	9.17E-04	4.86E-05	4.28E-05
Standard Deviation	6.74E-05	3.07E-03	1.04E-01	5.57E-02	1.14E-04	1.58E-04
Percent Standard Deviation (1σ)	89.9	187.4	223.2	180.3	120.1	143.1

Table S3. Mass balance and organism estimation model of sodium at Aşıklı Höyük.

Defined Temporal Subsections of Middens at Aşıklı Höyük			Refuse (Average)	Refuse (Level 2)	Refuse (Level 3)	Refuse (Level 4)	Refuse (Level 5)	Reference
Archaeological Midden Sodium Concentration	**Symbol**	**Units**						
sodium concentration	C_{Na}	$g\ kg^{-1}$	0.7274	1.2825	1.1037	0.4328	0.0905	(1)
sodium concentration	C_{Na}	$mol\ kg^{-1}$	0.0316	0.0558	0.0480	0.0188	0.0039	(1)
midden density	ρ_{refuse}	$kg\ m^{-3}$	1400	1400	1400	1400	1400	(1)
Calculated Na in Midden	$C_{Tot\ Na}$	**($mol\ m^{-3}$)**	**44.29**	**78.10**	**67.21**	**26.36**	**5.51**	
**Wood Ash**								
sodium concentration	$C_{ash\ Na}$	$g\ kg^{-1}$	3.4	3.4	3.4	3.4	3.4	(2,3)
sodium concentration	$C_{ash\ Na}$	$mol\ kg^{-1}$	0.148	0.148	0.148	0.148	0.148	(2,3)
wood ash density	ρ_{ash}	$kg\ m^{-3}$	760	760	760	760	760	(4)
fraction of midden	F_{ash}		0.25	0.25	0.25	0.25	0.25	(1)
Calculated Na in Wood Ash	$C_{Tot\ ash\ Na}$	**($mol\ m^{-3}$)**	**28.10**	**28.10**	**28.10**	**28.10**	**28.10**	
**Construction Debris**								
sodium concentration	$C_{cd\ Na}$	$g\ kg^{-1}$	0.0826	0.0826	0.0826	0.0826	0.0826	(1)
sodium concentration	$C_{cd\ Na}$	$mol\ kg^{-1}$	0.0036	0.0036	0.0036	0.0036	0.0036	(1)
construction debris density	ρ_{cd}	$kg\ m^{-3}$	1400	1400	1400	1400	1400	(1)
fraction of midden	F_{cd}		0.71	0.71	0.71	0.71	0.71	(1)
Calculated Na in Construction Debris	$C_{Tot\ cd\ Na}$	**($mol\ m^{-3}$)**	**3.57**	**3.57**	**3.57**	**3.57**	**3.57**	
**Rainfall**								
sodium concentration	$C_{rain\ Na}$	$g\ L^{-1}$	0.0002	0.0002	0.0002	0.0002	0.0002	(5)
sodium concentration	$C_{rain\ Na}$	$mol\ L^{-1}$	8.70E-06	8.70E-06	8.70E-06	8.70E-06	8.70E-06	(5)
runoff ratio	α		0.1	0.1	0.1	0.1	0.1	(6,7,8)
annual rainfall	R	$L\ m^{-2}\ yr^{-1}$	400	400	400	400	400	(9)
time since initial occupation	t	yr	10000	10000	10000	10000	10000	(10)
total flux of sodium from rain since occupation	$\Phi_{rain\ final\ Na}$	$mol\ m^{-2}$	31.32	31.32	31.32	31.32	31.32	(1)
top of the mound rainfall sodium concentration	$\Phi_{rain\ o\ Na}$	$mol\ m^{-2}$	23.49	23.49	23.49	23.49	23.49	(1)
constant for rainfall exponential function	k	m^{-1}	-0.75	-0.75	-0.75	-0.75	-0.75	(1)
top of level	h_T	m	N/A	0	5	9	12	(10)
bottom of level	h_B	m	N/A	5	9	12	14	(10)
total flux of sodium per level since occupation	$\Phi_{rain\ level\ Na}$	$mol\ m^{-2}$	N/A	30.58	0.70	0.03	0.00	(1)
Calculated Na in Rainfall	$C_{Tot\ rain\ Na}$	**($mol\ m^{-3}$)**	**1.58**	**6.12**	**0.17**	**0.01**	**0.00**	
**Residual**								
Calculated Na in Residual	$C_{residual\ Na}$	**($mol\ m^{-3}$)**	**11.05**	**40.31**	**35.36**	**-5.33**	**-26.16**	
**Population Density Estimates**								
sodium concentration (average of human and caprine)	$C_{urine\ Na}$	$g\ L^{-1}\ org^{-1}$	0.5585	0.5585	0.5585	0.5585	0.5585	(11,12,13)
sodium concentration (average of human and caprine)	$C_{urine\ Na}$	$mol\ L^{-1}\ org^{-1}$	0.0243	0.0243	0.0243	0.0243	0.0243	(11,12,13)
runoff ratio	α		0	0	0	0	0	(1)
urination rate	U_R	$L\ yr^{-1}$	547.9	547.9	547.9	547.9	547.9	(14,15,16)
fraction of time spent on mound	F_{om}		0.5	0.5	0.5	0.5	0.5	(1)
sedimentation rate (constant)	Γ	$m\ yr^{-1}$	0.0165	0.0165	0.0165	0.0165	0.0165	(10)
sedimentation rate (variable)	Γ	$m\ yr^{-1}$	N/A	0.025	0.015	0.0033	0.0165	(10)
Calculated Population Density w/ Constant Sed. Rate	$D_{org\ i}$	**$org\ m^{-2}$**	**0.027**	**0.100**	**0.088**	**-0.013**	**-0.065**	
Caclulated Population Density w/ Variable Sed. Rate	$D_{org\ i}$	**$org\ m^{-2}$**	**N/A**	**0.151**	**0.080**	**-0.003**	**-0.065**	
**Total Population Estimates**								
tell area	A	m^2	57,227	57,227	57,227	57,227	57,227	(1)
Calculated Population w/ Constant Sed. Rate	$N_{org\ i}$	**org**	**1567.4**	**5719.6**	**5017.5**	**-755.7**	**-3711.8**	
Caclulated Population w/ Variable Sed. Rate	$N_{org\ i}$	**org**	**N/A**	**8666.1**	**4561.3**	**-151.1**	**-3711.8**	

References

(1) This study
(2) Etiégni and Campbell, 1991
(3) Demeyer et al., 2000
(4) Abdullahi, 2006
(5) Berner and Berner, 1987
(6) Coombs and Melack, 2013
(7) Cerda, 1998
(8) Cerda et al., 1998
(9) Türkeş, 1996
(10) Quade et al., 2018
(11) Karak and Bhattacharyya, 2011
(12) Kirchmann and Petterson, 1995
(13) Shand et al., 2002
(14) Guyton, 1986
(15) Frame, 1971
(16) Wolgast, 1993

Table S4. Mass balance and organism estimation model of chlorine at Aşıklı Höyük.

Defined Temporal Subsections of Middens at Aşıklı Höyük			Refuse (Average)	Refuse (Level 2)	Refuse (Level 3)	Refuse (Level 4)	Refuse (Level 5)	Reference
Archaeological Midden Chlorine Concentration	**Symbol**	**Units**						
chlorine concentration	C_{Cl}	g kg^{-1}	1.4482	1.9897	3.0455	0.6949	0.0629	(1)
chlorine concentration	C_{Cl}	mol kg^{-1}	0.0409	0.0561	0.0859	0.0196	0.0018	(1)
midden density	ρ_{refuse}	kg m^{-3}	1400	1400	1400	1400	1400	(1)
Calculated Cl in Midden	$C_{Tot\ Cl}$	**(mol m^{-3})**	**57.19**	**78.58**	**120.27**	**27.44**	**2.48**	
**Wood Ash**								
chlorine concentration	$C_{ash\ Cl}$	g kg^{-1}	0.0010	0.0010	0.0010	0.0010	0.0010	(2)
chlorine concentration	$C_{ash\ Cl}$	mol kg^{-1}	2.82E-05	2.82E-05	2.82E-05	2.82E-05	2.82E-05	(2)
wood ash density	ρ_{ash}	kg m^{-3}	760	760	760	760	760	(3)
fraction of midden	F_{ash}		0.25	0.25	0.25	0.25	0.25	(1)
Calculated Cl in Wood Ash	$C_{Tot\ ash\ Cl}$	**(mol m^{-3})**	**0.01**	**0.01**	**0.01**	**0.01**	**0.01**	
**Construction Debris**								
chlorine concentration	$C_{cd\ Cl}$	g kg^{-1}	0.0643	0.0643	0.0643	0.0643	0.0643	(1)
chlorine concentration	$C_{cd\ Cl}$	mol kg^{-1}	0.0018	0.0018	0.0018	0.0018	0.0018	(1)
construction debris density	ρ_{cd}	kg m^{-3}	1400	1400	1400	1400	1400	(1)
fraction of midden	F_{cd}		0.71	0.71	0.71	0.71	0.71	(1)
Calculated Cl in Construction Debris	$C_{Tot\ cd\ Cl}$	**(mol m^{-3})**	**1.80**	**1.80**	**1.80**	**1.80**	**1.80**	
**Rainfall**								
chlorine concentration	$C_{rain\ Cl}$	g L^{-1}	0.0002	0.0002	0.0002	0.0002	0.0002	(4)
chlorine concentration	$C_{rain\ Cl}$	mol L^{-1}	5.64E-06	5.64E-06	5.64E-06	5.64E-06	5.64E-06	(4)
runoff ratio	α		0.1	0.1	0.1	0.1	0.1	(5,6,7)
annual rainfall	R	L m^{-2} yr^{-1}	400	400	400	400	400	(8)
time since initial occupation	t	yr	10000	10000	10000	10000	10000	(9)
total flux of chlorine from rain since occupation	$\Phi_{rain\ final\ Cl}$	mol m^{-2}	20.31	20.31	20.31	20.31	20.31	(1)
top of the mound rainfall chlorine concentration	$\Phi_{rain\ o\ Cl}$	mol m^{-2}	15.23	15.23	15.23	15.23	15.23	(1)
constant for rainfall exponential function	k	m^{-1}	-0.75	-0.75	-0.75	-0.75	-0.75	(1)
top of level	h_T	m	N/A	0	5	9	12	(9)
bottom of level	h_B	m	N/A	5	9	12	14	(9)
total flux of chlorine per level since occupation	$\Phi_{rain\ level\ Cl}$	mol m^{-2}	N/A	19.83	0.45	0.02	0.00	(1)
Calculated Cl in Rainfall	$C_{Tot\ rain\ Cl}$	**(mol m^{-3})**	**1.02**	**3.97**	**0.11**	**0.01**	**0.00**	
**Residual**								
Calculated Cl in Residual	$C_{residual\ Cl}$	**(mol m^{-3})**	**54.37**	**72.80**	**118.35**	**25.63**	**0.67**	
**Population Density Estimates**								
chlorine concentration (average of human and caprine)	$C_{urine\ Cl}$	g L^{-1} org^{-1}	2.7938	2.7938	2.7938	2.7938	2.7938	
chlorine concentration (average of human and caprine)	$C_{urine\ Cl}$	mol L^{-1} org^{-1}	0.0788	0.0788	0.0788	0.0788	0.0788	(10,11,12,13)
runoff ratio	α		0	0	0	0	0	
urination rate	U_R	L yr^{-1}	547.9	547.9	547.9	547.9	547.9	(14,15,16)
fraction of time spent on mound	F_{om}		0.5	0.5	0.5	0.5	0.5	(1)
sedimentation rate (constant)	Γ	m yr^{-1}	0.0165	0.0165	0.0165	0.0165	0.0165	(9)
sedimentation rate (variable)	Γ	m yr^{-1}	N/A	0.025	0.015	0.0033	0.0165	(9)
Calculated Population Density w/ Constant Sed. Rate	$D_{org\ i}$	**org m^{-2}**	**0.042**	**0.056**	**0.090**	**0.020**	**0.001**	
Caclulated Population Density w/ Variable Sed. Rate	$D_{org\ i}$	**org m^{-2}**	**N/A**	**0.084**	**0.082**	**0.004**	**0.001**	
**Total Population Estimates**								
tell area	A	m^2	57,227	57,227	57,227	57,227	57,227	(1)
Calculated Population w/ Constant Sed. Rate	$N_{org\ i}$	**org**	**2377.8**	**3184.4**	**5176.6**	**1121.0**	**29.5**	
Caclulated Population w/ Variable Sed. Rate	$N_{org\ i}$	**org**	**N/A**	**4824.8**	**4706.0**	**224.2**	**29.5**	

References
(1) This study
(2) Thy et al., 2006
(3) Abdullahi, 2006
(4) Berner and Berner, 1987
(5) Coombs and Melack, 2013
(6) Cerda, 1998
(7) Cerda et al., 1998
(8) Türkeş, 1996
(9) Quade et al., 2018
(10) Karak and Bhattacharyya, 2011
(11) Kirchmann and Petterson, 1995
(12) Haynes and Williams, 1993
(13) Shand et al., 2002
(14) Guyton, 1986
(15) Frame, 1971
(16) Wolgast, 1993

Table S5. Mass balance and organism estimation model of nitrate at Aşıklı Höyük.

Defined Temporal Subsections of Middens at Aşıklı Höyük			Refuse (Average)	Refuse (Level 2)	Refuse (Level 3)	Refuse (Level 4)	Refuse (Level 5)	Reference
Archaeological Midden Nitrate Concentration	**Symbol**	**Units**						
nitrate concentration	C_{NO3}	g kg^{-1}	1.2274	1.9143	2.8891	0.1017	0.0047	(1)
nitrate concentration	C_{NO3}	mol kg^{-1}	0.0198	0.0309	0.0466	0.0016	0.0001	(1)
midden density	ρ_{refuse}	kg m^{-3}	1400	1400	1400	1400	1400	(1)
Calculated NO3 in Midden	$C_{Tot\ NO3}$	(mol m^{-3})	**27.71**	**43.22**	**65.23**	**2.30**	**0.10**	
**Wood Ash**								
nitrate concentration	$C_{ash\ NO3}$	g kg^{-1}	0.6	0.6	0.6	0.6	0.6	(2,3)
nitrate concentration	$C_{ash\ NO3}$	mol kg^{-1}	0.010	0.010	0.010	0.010	0.010	(2,3)
wood ash density	ρ_{ash}	kg m^{-3}	760	760	760	760	760	(4)
fraction of midden	F_{ash}		0.25	0.25	0.25	0.25	0.25	(1)
Calculated NO3 in Wood Ash	$C_{Tot\ ash\ NO3}$	(mol m^{-3})	**1.84**	**1.84**	**1.84**	**1.84**	**1.84**	
**Construction Debris**								
nitrate concentration	$C_{cd\ NO3}$	g kg^{-1}	0.0059	0.0059	0.0059	0.0059	0.0059	(1)
nitrate concentration	$C_{cd\ NO3}$	mol kg^{-1}	0.0001	0.0001	0.0001	0.0001	0.0001	(1)
construction debris density	ρ_{cd}	kg m^{-3}	1400	1400	1400	1400	1400	(1)
fraction of midden	F_{cd}		0.71	0.71	0.71	0.71	0.71	(1)
Calculated NO3 in Construction Debris	$C_{Tot\ cd\ NO3}$	(mol m^{-3})	**0.09**	**0.09**	**0.09**	**0.09**	**0.09**	
**Rainfall**								
nitrate concentration	$C_{rain\ NO3}$	g L^{-1}	0.0005	0.0005	0.0005	0.0005	0.0005	(5)
nitrate concentration	$C_{rain\ NO3}$	mol L^{-1}	8.06E-06	8.06E-06	8.06E-06	8.06E-06	8.06E-06	(5)
runoff ratio	α		0.1	0.1	0.1	0.1	0.1	(6,7,8)
annual rainfall	R	L m^{-2} yr^{-1}	400	400	400	400	400	(9)
time since initial occupation	t	yr	10000	10000	10000	10000	10000	(10)
total flux of nitrate from rain since occupation	$\Phi_{rain\ final\ NO3}$	mol m^{-2}	29.03	29.03	29.03	29.03	29.03	(1)
top of the mound rainfall nitrate concentration	$\Phi_{rain\ o\ NO3}$	mol m^{-2}	21.77	21.77	21.77	21.77	21.77	(1)
constant for rainfall exponential function	k	m^{-1}	-0.75	-0.75	-0.75	-0.75	-0.75	(1)
top of level	h_T	m	N/A	0	5	9	12	(10)
bottom of level	h_B	m	N/A	5	9	12	14	(10)
total flux of nitrate per level since occupation	$\Phi_{rain\ level\ NO3}$	mol m^{-2}	N/A	28.35	0.65	0.03	0.00	(1)
Calculated NO3 in Rainfall	$C_{Tot\ rain\ NO3}$	(mol m^{-3})	**1.46**	**5.67**	**0.16**	**0.01**	**0.00**	
**Residual**								
Calculated NO3 in Residual	$C_{residual\ NO3}$	(mol m^{-3})	**24.32**	**35.62**	**63.13**	**0.35**	**-1.83**	
**Population Density Estimates**								
nitrogen concentration (average of human and caprine)	$C_{urine\ NO3}$	g L^{-1} org^{-1}	6.605	6.605	6.605	6.605	6.605	(13,14,15,16,17)
fraction ammonia volatilization remaining	F_{av}		0.55	0.55	0.55	0.55	0.55	(11,12)
nitrate concentration (average of human and caprine)	$C_{urine\ NO3}$	mol L^{-1} org^{-1}	0.0586	0.0586	0.0586	0.0586	0.0586	(13,14,15,16,17)
runoff ratio	α		0	0	0	0	0	(1)
urination rate	U_R	L yr^{-1}	547.9	547.9	547.9	547.9	547.9	(15,18,19)
fraction of time spent on mound	F_{om}		0.5	0.5	0.5	0.5	0.5	(1)
sedimentation rate (constant)	Γ	m yr^{-1}	0.0165	0.0165	0.0165	0.0165	0.0165	(10)
sedimentation rate (variable)	Γ	m yr^{-1}	N/A	0.025	0.015	0.0033	0.0165	(10)
Calculated Population Density w/ Constant Sed. Rate	$D_{org\ i}$	**org m^{-2}**	**0.025**	**0.037**	**0.065**	**0.000**	**-0.002**	
Caclulated Population Density w/ Variable Sed. Rate	$D_{org\ i}$	**org m^{-2}**	**N/A**	**0.055**	**0.059**	**0.000**	**-0.002**	
**Total Population Estimates**								
tell area	A	m^{2}	57,227	57,227	57,227	57,227	57,227	(1)
Calculated Population w/ Constant Sed. Rate	$N_{org\ i}$	**org**	**1430.8**	**2095.7**	**3714.6**	**20.7**	**-107.6**	
Caclulated Population w/ Variable Sed. Rate	$N_{org\ i}$	**org**	**N/A**	**3175.2**	**3376.9**	**4.1**	**-107.6**	

References

(1) This study
(2) Etiégni and Campbell, 1991
(3) Demeyer et al., 2000
(4) Abdullahi, 2006
(5) Berner and Berner, 1987
(6) Coombs and Melack, 2013
(7) Cerda, 1998
(8) Cerda et al., 1998
(9) Türkeş, 1996
(10) Quade et al., 2018
(11) Reynolds and Wolf , 1987
(12) Whitehead and Raistrick, 1992
(13) Karak and Bhattacharyya, 2011
(14) Pradhan et al., 2009
(15) Guyton, 1986
(16) Ban and Dave, 2004
(17) Shand et al., 2002
(18) Frame, 1971
(19) Wolgast, 1993

Table S6. Density data from midden, construction material, and alluvium samples at Aşıklı Höyük.

Sample Number	Sample Type	Mass (g)	Volume Difference (cm^3)	Density ($g\ cm^{-3}$)
AHJQ - 1110	Bulk Midden	1.1	0.9	1.2
AHJQ - 1113	Bulk Midden	1.2	1.1	1.1
AHJQ - 1097	Plaster	1.4	1.2	1.2
AHJQ - 1108	Bulk Midden	1.5	1.4	1.1
AHJQ - 1083	Bulk Soil	1.3	1	1.3
AHJQ - 1138	Alleyway	1.5	1.2	1.3
AHJQ - 1139	Alleyway	1.7	1.3	1.3
AHJQ - 1116	Bulk Midden	1.5	1.2	1.3
AHJQ - 1095-1	Bulk Soil	1.9	1.3	1.5
AHJQ - 1111	Bulk Midden	1.9	1.4	1.4
AHJQ - 1176	Bulk Midden	1.6	1	1.6
AHJQ - 1174	Bulk Midden	1.4	0.8	1.8
AHJQ - 1133	Bulk Midden	0.9	0.8	1.1
AHJQ - 1127	Bulk Midden	1.4	1.1	1.3
AHJQ - 1096	Plaster	1.7	1	1.7
AHJQ - 1153	Charcoal and Hearth Material	1.8	0.9	2.0
AHJQ - 1131	Bulk Midden	1.3	0.8	1.6
AHJQ - 1146	Alleyway	1.2	1	1.2
AHJQ - 1168	Bulk Midden	1.8	1	1.8
AHJQ - 1084	Bulk Midden	1.1	0.9	1.2
	**Midden Average**			**1.4**
	**Construction Debris Average**			**1.4**
